# Upregulation of KLK8 contributes to CUMS-induced hippocampal neuronal apoptosis by cleaving NCAM1

**DOI:** 10.1038/s41419-023-05800-5

**Published:** 2023-04-19

**Authors:** Dan-Hong Xu, Jian-Kui Du, Shi-Yu Liu, Hui Zhang, Lu Yang, Xiao-Yan Zhu, Yu-Jian Liu

**Affiliations:** 1 School of Kinesiology, Shanghai Frontiers Science Research Base of Exercise and Metabolic Health, The Key Laboratory of Exercise and Health Sciences of Ministry of Education Shanghai University of Sport, Shanghai, 200438 China; 2 Department of Physiology, Navy Medical University, Shanghai, 200433 China; 3 National Clinical Research Center for Geriatric Disorders and National International Joint Research Center for Medical Metabolomics, Xiangya Hospital, Central South University, Changsha, Hunan, 41008 China; 4 Department of Anesthesiology and Surgical Intensive Care Unit, Xinhua Hospital, Shanghai Jiaotong University School of Medicine, Shanghai, 200092 China

**Keywords:** Cell death in the nervous system, Neurophysiology

## Abstract

Neuronal apoptosis has been well-recognized as a critical mediator in the pathogenesis of depressive disorders. Tissue kallikrein-related peptidase 8 (KLK8), a trypsin-like serine protease, has been implicated in the pathogenesis of several psychiatric disorders. The present study aimed to explore the potential function of KLK8 in hippocampal neuronal cell apoptosis associated with depressive disorders in rodent models of chronic unpredictable mild stress (CUMS)-induced depression. It was found that depression-like behavior in CUMS-induced mice was associated with hippocampal KLK8 upregulation. Transgenic overexpression of KLK8 exacerbated, whereas KLK8 deficiency attenuated CUMS-induced depression-like behaviors and hippocampal neuronal apoptosis. In HT22 murine hippocampal neuronal cells and primary hippocampal neurons, adenovirus-mediated overexpression of KLK8 (Ad-KLK8) was sufficient to induce neuron apoptosis. Mechanistically, it was identified that the neural cell adhesion molecule 1 (NCAM1) may associate with KLK8 in hippocampal neurons as KLK8 proteolytically cleaved the NCAM1 extracellular domain. Immunofluorescent staining exhibited decreased NCAM1 in hippocampal sections obtained from mice or rats exposed to CUMS. Transgenic overexpression of KLK8 exacerbated, whereas KLK8 deficiency largely prevented CUMS-induced loss of NCAM1 in the hippocampus. Both adenovirus-mediated overexpression of NCAM1 and NCAM1 mimetic peptide rescued KLK8-overexpressed neuron cells from apoptosis. Collectively, this study identified a new pro-apoptotic mechanism in the hippocampus during the pathogenesis of CUMS-induced depression via the upregulation of KLK8, and raised the possibility of KLK8 as a potential therapeutic target for depression.

## Introduction

Depression is considered a critical mental disorder associated with altered neuronal structure and function in specific brain regions [1–3], which contributes to severe mental symptoms, high suicidal tendency, and a heavy burden on society and individuals [4]. As a major form of stress, chronic stress causes numerous impairments in mood, cognition, and memory, and is considered a risk factor for depressive disorders [5, 6]. In dysregulated stress responses, environmental stressors cause stable changes in gene expression, dysregulation of neurotransmitters and cytokines, which may lead to neuronal apoptosis and altered hippocampal neurogenesis [6–9]. Growing evidence has established the association between stressful life events and depression, yet the molecular mechanisms by which stress induces depression is not well understood.

Apoptosis is a process that is tightly controlled by pro-apoptotic and anti-apoptotic signaling pathways that play a central role in controlling cell numbers and tissue size to maintain normal structural and functional homeostasis [10, 11]. Apoptotic cells have been found in the hippocampus of major depressed patients [12, 13]. Animal studies demonstrate that neuronal apoptosis contributes to structural hippocampal changes in the pathogenesis of depression-like behaviors from different etiologies, including diabetes-related depression and chronic stress-induced depression [14–16]. Controlling apoptosis is therefore expected to hold great potential for the treatment of depressive disorders.

Tissue kallikrein-related peptidase 8 (KLK8), also named neuropsin, is a trypsin-like serine protease expressed abundantly in the central nervous system (CNS) [17]. KLK8 has been implicated in the pathogenesis of several psychiatric disorders, such as anxiety, depression, and schizophrenia [17, 18]. Recently, Starnawska et al. identified an association between blood DNA methylation levels in promoter regions of KLK8 and severity of depression symptoms in a large Danish cohort of monozygotic twins [19] and four independent methylomic cohorts [20], further supporting the implication of KLK8 in depression symptomatology. Mechanically, the KLK8-mediated cleavage of ephrin type-B receptor 2 is involved in the pathogenesis of anxiety disorders [21]. Intriguingly, our group recently reported that KLK8-mediated cleavage of VE-cadherin, a major adhesive protein of inter-endothelial junctions, contributes to hyperglycemia-induced impairment of endothelial cell viability [22]. These findings prompted us to explore the potential function of KLK8 in hippocampal neuronal cell apoptosis associated with depressive disorders.

In the present study, we first examined the hippocampal expression of KLK8 in a mouse model of chronic unpredictable mild stress (CUMS)-induced depression. Using both KLK8 knockout mice and KLK8 transgenic rats, the present study investigated the precise role of KLK8 in mediating CUMS-induced hippocampal neuronal apoptosis and depression-like behavior. In addition, the molecular mechanisms underlying KLK8-mediated neuronal apoptosis were further illustrated.

## Materials and methods

### Animals

All laboratory mice and rats in this study were maintained in a pathogen-free facility at the Animal Research Center of Navy Medical University. Animal studies were performed in accordance with the Guide for the Care and Use of Laboratory Animals published by the NIH (NIH publication No. 85-23, revised 1996), and were approved by the Ethical Committee of Experimental Animals of Shanghai University of Sport. Details were described in the supplemental materials and methods.

### Chronic unpredictable mild stress (CUMS)

Details for the CUMS procedure were described in the supplemental materials and methods.

### Behavioral measurements

Depressive-like phenotypes in rodent models were validated by behavioral measurements, including sucrose preference test (SPT), novelty-suppressed feeding test (NSFT), forced swimming test (FST), and tail-suspension test (TST). Details were described in the supplemental materials and methods.

### Cell culture and adenoviral infection

HT22 murine hippocampal neuronal cells and hippocampal primary isolated neuron cells were used in this study and infected with KLK8 adenovirus. Details were described in the supplemental materials and methods.

### Cell viability assays

Cell viability was assayed using cell counting kit-8, and the detailed protocols were described in the supplemental materials and methods.

### Measurement of caspase-3 activity

Caspase-3 activity was measured in the HT22 cells, and the detailed protocols were described in the supplemental materials and methods.

### Immunofluorescence

The hippocampal cryosections were cut in a cryostat and processed for immunofluorescence. The detailed protocols were described in the supplemental materials and methods.

### TdT-mediated dUTP nick-end labeling (TUNEL) assay

The hippocampal cryosections and HT22 cells were stained using the One Step TUNEL Apoptosis Assay Kit, and the detailed protocols were described in the supplemental materials and methods.

### Western blot and immunoprecipitation

The hippocampus tissue and HT22 cell lysates were used for western blot and immunoprecipitation assays. The detailed protocols were described in the supplemental materials and methods.

### Mass Spectrometry

Proteins extracted from the hippocampal tissue of KLK8 transgenic rats were immunoprecipitated with primary antibodies against KLK8. The immunoprecipitates were separated by SDS-PAGE. Protein sections from the SDS-PAGE gel were digested with trypsin, and then identified by ultra-performance liquid chromatography-tandem mass spectrometry (UPLC-MS/MS). The detailed protocols were described in the supplemental materials and methods.

### N-terminal protein sequencing

N-terminal sequencing was performed to identify the aminoterminal sequence of the extracellular fragment of the neural cell adhesion molecule 1 (NCAM1) released into the culture medium of KLK8-overexpressed HT22 cells. The detailed protocols were described in the supplemental materials and methods.

### Statistical analysis

The statistical analysis was performed using SPSS 22.0 (SPSS Inc., Chicago, USA). The analysis methods were described in the supplemental materials and methods.

## Results

### Depression-like behaviors in CUMS-exposed mice are associated with hippocampal KLK8 upregulation

**Fig. 1:**
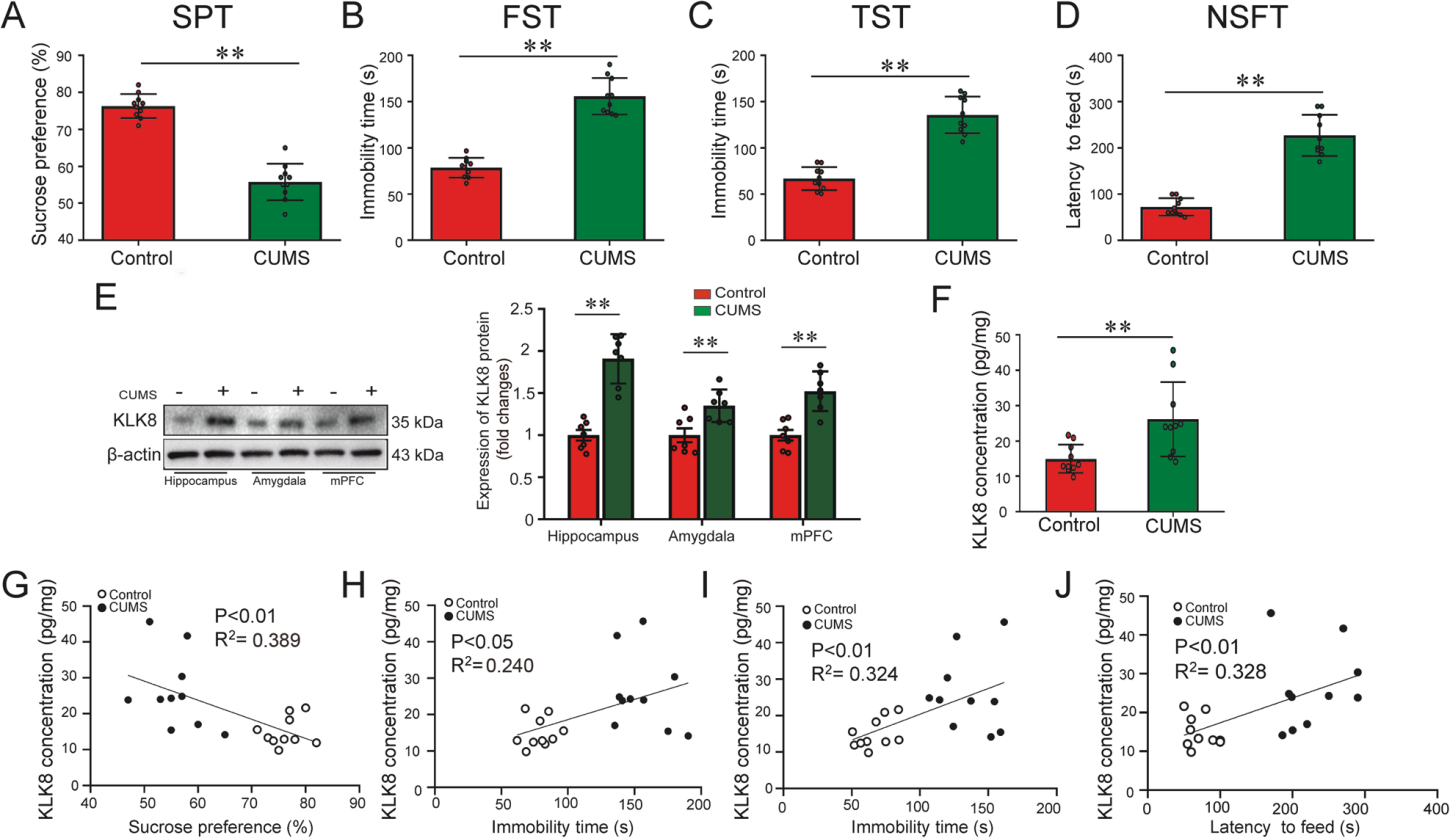
Depression-like behaviors in CUMS-exposed mice are associated with hippocampal KLK8 upregulation. **A**–**D** Depressive behavioral tests were performed in mice exposed to chronic unpredictable mild stress (CUMS) for 5 weeks (*n* = 10, unpaired *t*-test). **A** Indicated the sucrose preference test (SPT). **B** Indicated the immobility time in the forced swimming test (FST). **C** Indicated the immobility time in the tail-suspension test (TST). **D** Indicated the latency to feed in the novelty-suppressed feeding test (NSFT). **E** The levels of KLK8 protein were measured in the hippocampus, amygdala, and medial prefrontal cortex (mPFC) of mice exposed to CUMS for 5 weeks by western blot analysis. Representative protein bands were presented on the left of the histograms (*n* = 7, unpaired *t*-test). **F** The levels of KLK8 protein were measured in the hippocampus of mice exposed to CUMS for 5 weeks by ELISA assay (*n* = 10, unpaired *t*-test). **G**–**J** Showed the correlations between hippocampal KLK8 protein levels determined by ELISA assay and the sucrose preference (**G**), the immobility times in FST (**H**), the immobility times in TST (**I**), and the latency to feed in the NSFT (**J**) (*n* = 10, Pearson correlation). Data were presented as means ± SD. **p < 0.01.

Previous studies have implicated KLK8 in the pathogenesis of several psychiatric disorders, including anxiety and depression [17, 18]. In the present study, we first examined whether the depressive phenotypes of CUMS-exposed mice are associated with hippocampus KLK8 expression. Depressive phenotypes of CUMS-exposed mice were evaluated in the fifth week of the study by sucrose preference test (SPT), tail-suspension test (TST), forced swimming test (FST), and novelty-suppressed feeding test (NSFT). As shown in Fig. 1A, 5 weeks CUMS-exposed mice exhibited a significant reduction of sucrose preference, which indicated anhedonia and impaired sensitivity to reward. In addition, CUMS-exposed mice exhibited longer immobility times in the FST (Fig. 1B) and TST (Fig. 1C), as well as an increased latency to feed in the NSFT (Fig. 1D), as compared to control mice.

Several brain regions, including the hippocampus, medial prefrontal cortex (mPFC), and amygdala, are involved in CUMS-induced depression-like behaviors [23]. As shown in Fig. 1E, it was found that 5 weeks CUMS-exposed mice exhibited significantly increased KLK8 expression in the hippocampus, mPFC, and amygdala as compared to control mice. Notably, KLK8 showed the highest upregulation in the hippocampus compared to mPFC and amygdala. ELISA assays further confirmed the increases in hippocampal KLK8 protein levels in CUMS-exposed mice (Fig. 1F). As shown in Fig. 1G–J, we found a negative correlation between the sucrose preference and hippocampal levels of KLK8 (p < 0.01, correlation coefficient *r* = −0.624, *R*^2^ = 0.389). In contrast, the immobility times in the FST and TST were positively correlated with hippocampal levels of KLK8 (*p* < 0.05, the correlation coefficient *r* = 0.490, *R*^2^ = 0.240 for FST; *p* < 0.01, the correlation coefficient *r* = 0.569, *R*^2^ = 0.324 for TST). A significant positive correlation was also observed between the latency to feed in the NSFT and hippocampal KLK8 levels (*p* < 0.01, correlation coefficient *r* = 0.573, *R*^2^ = 0.328). These findings indicated that the depressive phenotypes of CUMS-exposed mice were associated with hippocampal KLK8 upregulation.

### Transgenic overexpression of KLK8 exacerbates, whereas KLK8 deficiency attenuates CUMS-induced depression-like behaviors

**Fig. 2:**
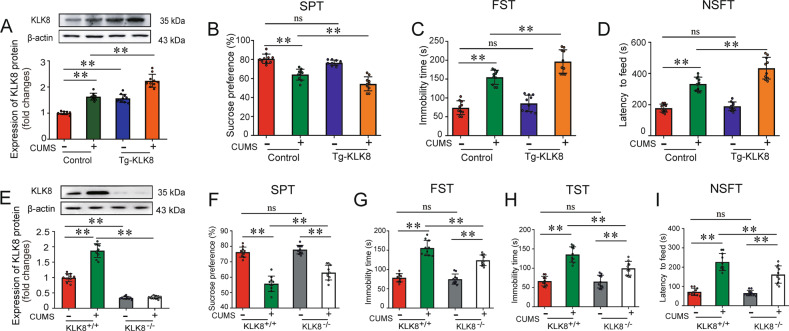
Transgenic overexpression of KLK8 exacerbates, whereas KLK8 deficiency attenuates CUMS-induced depression-like behaviors. KLK8 transgenic (Tg-KLK8) rats or KLK8-deficient (KLK8^−/−^) mice were exposed to CUMS for 5 weeks. **A**, **E** Protein levels of KLK8 in the hippocampus of Tg-KLK8 rats (**A**) or KLK8^−/−^ mice (**E**) were determined by western blot analysis. Representative protein bands were presented on the top of the histograms. **B**–**D** Depressive behavioral tests were performed in CUMS-exposed control and Tg-KLK8 rats. **B** Indicated the SPT. **C** Indicated the immobility time in the FST. **D** Indicated the latency to feed in the NSFT. **F**–**I** Depressive behavioral tests were performed in CUMS-exposed wild-type and KLK8-deficient mice. **F** Indicated the SPT. **G** Indicated the immobility time in the FST. **H** Indicated the immobility time in the TST. **I** Indicated the latency to feed in the NSFT. Data were presented as means ± SD (*n* = 10, two-way ANOVA, Bonferroni’s post hoc test). **p < 0.01, ns not significant.

Using KLK8 transgenic rats, the present study first examined the effect of KLK8 overexpression on CUMS-induced depression-like behaviors. As shown in Fig. 2A, the CUMS-induced upregulation of hippocampal KLK8 was further enhanced in KLK8 transgenic rats. It was found that CUMS-exposed KLK8 transgenic rats displayed increased depression-like behavioral responses, as evidenced by decreased sucrose preference (Fig. 2B) and longer immobility time in the FST (Fig. 2C) and increased latency to feed in the NSFT (Fig. 2D), as compared to CUMS-exposed control rats. We also noticed that KLK8 transgenic rats displayed trends of reduced sucrose preference and increased immobility time in the FST as compared to control rats, albeit not statistically significant.

We then examined the CUMS-induced depression-like behaviors in KLK8-deficient mice. As shown in Fig. 2E, the CUMS-induced upregulation of hippocampal KLK8 was blunt in KLK8-deficient (KLK8^−/−^) mice. CUMS-exposed KLK8-deficient mice exhibited significant increases in the sucrose preference, compared with CUMS-exposed wild-type mice (Fig. 2F). Moreover, the CUMS-induced increases in the immobility times in FST (Fig. 2G) and TST (Fig. 2H), as well as increases in the latency to feed in the NSFT (Fig. 2I) were significantly decreased in KLK8-deficient mice.

Taken together, these results indicated that KLK8 overexpression was exacerbated, whereas KLK8 deficiency attenuated the depression-like behaviors in experimental rodent models of CUMS.

### Transgenic overexpression of KLK8 exacerbates, whereas KLK8 deficiency attenuates CUMS-induced neuron apoptosis

**Fig. 3:**
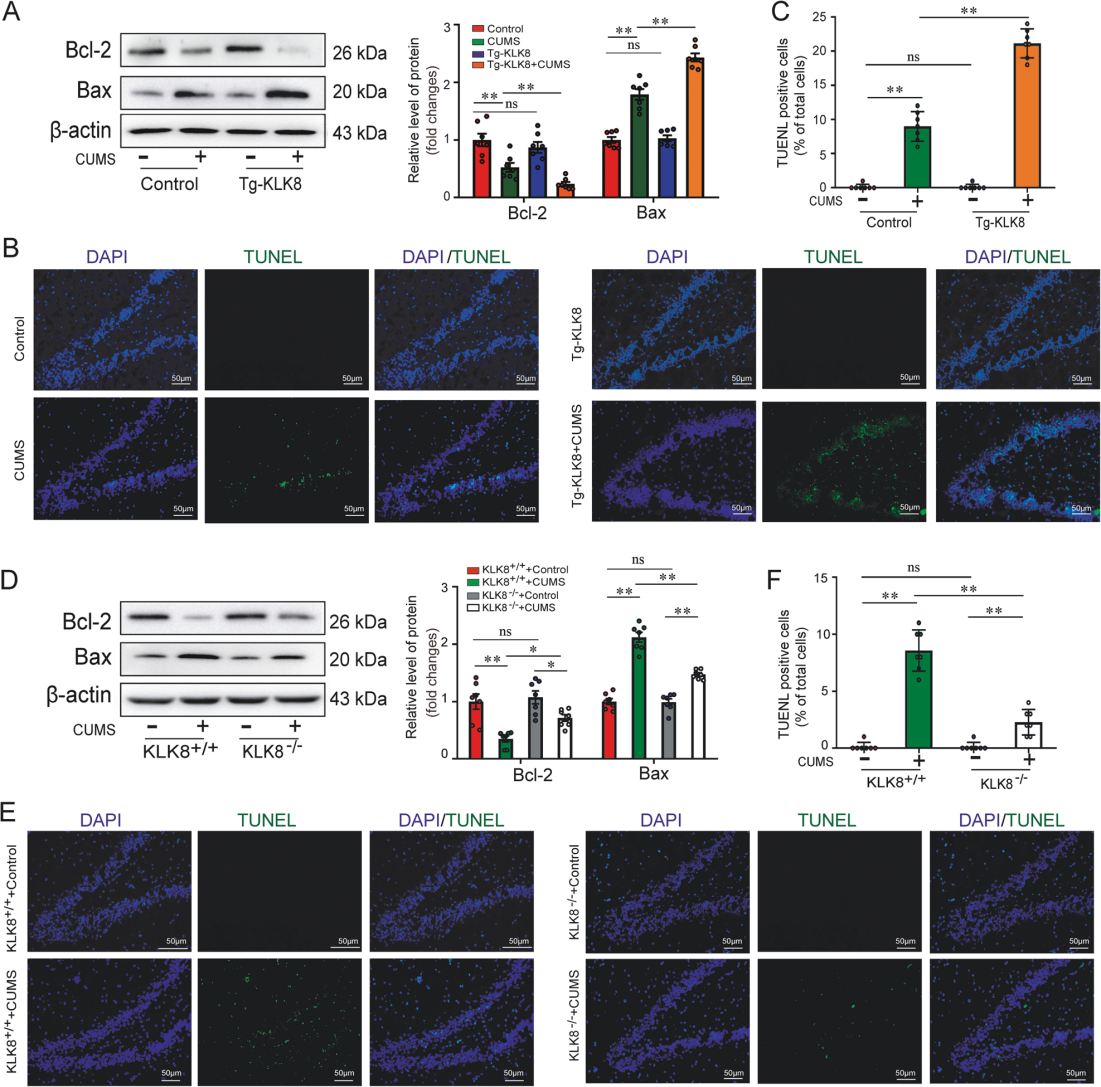
Transgenic overexpression of KLK8 exacerbates, whereas KLK8 deficiency attenuates CUMS-induced neuron apoptosis. KLK8 transgenic (Tg-KLK8) rats or KLK8-deficient (KLK8^-/-^) mice were exposed to CUMS for 5 weeks. **A**, **D** Protein levels of Bcl-2 and Bax in the hippocampus of Tg-KLK8 rats (**A**) or KLK8^−/−^ mice (**D**) were determined by western blot analysis. Representative protein bands were presented on the left of the histograms. **B**, **E** Showed representative TUNEL-stained cells (green) in the cryostat-cut hippocampal sections of Tg-KLK8 rats (**B**) or KLK8^−/−^ mice (**E**). Nuclei were counterstained with DAPI (blue). Scale bar = 50 μm. **C**, **F** The TUNEL-positive cell number (%) of Tg-KLK8 rats (**C**) or KLK8^−/−^ mice (**F**) was shown as a ratio of the number of TUNEL-positive cells to the total cell number. Data were presented as means ± SD (*n* = 7, two-way ANOVA, Bonferroni’s post hoc test). *p < 0.05, **p < 0.01, ns not significant.

Increased neuronal apoptosis in the hippocampus has been found to be associated with the depressive phenotype of chronically stressed animals [15, 16]. As shown in Fig. 3A, D, the anti-apoptotic protein Bcl-2 was decreased, whereas the pro-apoptotic protein Bax was increased in the hippocampus of CUMS-exposed rats/mice compared with the control rats/mice. DNA fragmentation, a characteristic of cell apoptosis, was then explored in hippocampal tissues using the TUNEL assay. As shown in Fig. 3B, C, E, F, the percentage of TUNEL-positive cells was significantly increased in the hippocampal tissue sections of CUMS-exposed rats/mice compared with the control rats/mice. These results provided evidence supporting a significant role of hippocampal neuron apoptosis in the pathogenesis of CUMS-induced depression-like behavior. In addition, the results demonstrated that transgenic KLK8 overexpression further increased Bax expression (Fig. 3A) and the percentage of TUNEL-positive cells (Fig. 3B, C), while the expressions of Bcl-2 (Fig. 3A) was notably decreased in the hippocampal tissues of CUMS-exposed rats. In contrast, KLK8 deficiency reduced Bax expression (Fig. 3D) and the percentage of TUNEL-positive cells (Fig. 3E, F), whereas it increased Bcl-2 protein expression (Fig. 3D) in the hippocampal tissues of CUMS-exposed mice. These results suggested that KLK8 upregulation contributed to hippocampal neuron apoptosis in CUMS-exposed animals.

Chronic stress-induced depression-like behaviors are also associated with neuron apoptosis in mPFC and the amygdala [24, 25]. We found that KLK8 deficiency significantly attenuated CUMS-induced apoptosis in mPFC (supplemental Fig. 1A–C) and amygdala (supplemental Fig. 2A–C), as evidenced by decreased Bax expression and TUNEL-positive cells, as well as increased Bcl-2 expression.

### KLK8 overexpression induces neuron apoptosis in vitro

**Fig. 4:**
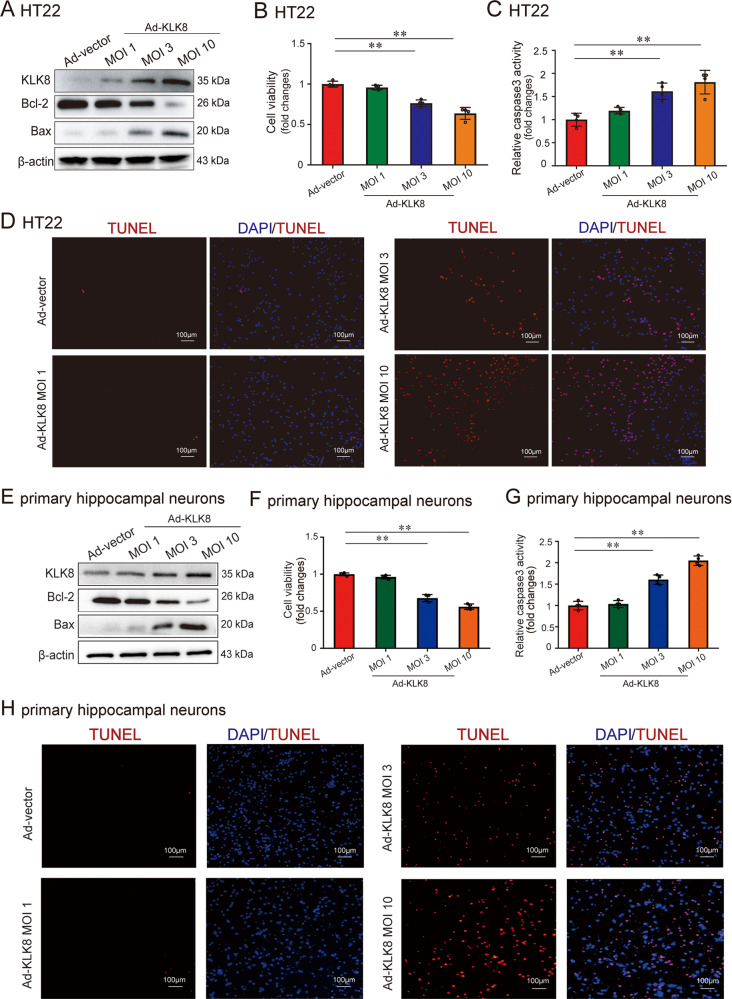
KLK8 overexpression induces neuron apoptosis in vitro. HT22 murine hippocampal neuronal cells (**A**–**D**) and primary hippocampal neurons (**E**–**H**) were infected with KLK8 adenovirus (Ad-KLK8) at a multiplicity of infection (MOI) of 1, 3, or 10 for 24 h. A, protein levels of KLK8, Bcl-2, and Bax in HT22 cells were determined by western blot analysis. **B**, **C** CCK8 assay and measurement of caspase-3 activity showed that Ad-KLK8 induced cell injury (**B**) and caspase-3 activation (**C**) in a dose-dependent manner in HT22 cells, respectively. **D** Showed representative TUNEL-stained (red) cells in HT22 cells. Nuclei were counterstained with DAPI (blue). Scale bar = 100 μm. **E** Protein levels of KLK8, Bcl-2, and Bax in primary hippocampal neurons were determined by western blot analysis. **F**, **G** CCK8 assay and measurement of caspase-3 activity showed that Ad-KLK8 induced cell injury (**F**) and caspase-3 activation (**G**) in a dose-dependent manner in primary hippocampal neurons, respectively. **H** Showed representative TUNEL-stained (red) cells in primary hippocampal neurons. Nuclei were counterstained with DAPI (blue). Scale bar = 100 μm. Data were presented as means ± SD (*n* = 4, one-way ANOVA, Bonferroni’s post hoc test). **p < 0.01.

The present study further confirmed the direct effect of KLK8 on hippocampal neurons. Infection of HT22 cells or primary isolated neonatal neurons with increasing concentrations of KLK8 adenovirus (Ad-KLK8) led to an increase in KLK8 expression in a dose-dependent manner (Fig. 4A, E and supplemental Fig. 3A, C). Cell damage of HT22 and primary isolated neonatal neurons occurred, as revealed by a decrease in cell viability (Fig. 4B, F). In addition, it was found that Ad-KLK8 treatment for 24 h dose-dependently increased caspase-3 activity (Fig. 4C, G), pro-apoptotic protein Bax expression (Fig. 4A, E and supplemental Fig. 3A, C) and the percentage of TUNEL-positive cells (Fig. 4D, H and supplemental Fig. 3B, D) in HT22 and primary isolated neonatal neurons. In contrast, anti-apoptotic protein Bcl-2 expression (Fig. 4A, E and supplemental Fig. 3A, C) was decreased after Ad-KLK8 treatment. These findings indicated that KLK8 overexpression was sufficient to induce hippocampal neuron apoptosis.

### NCAM1 may be recognized as a potential substrate of KLK8 in neuron

**Fig. 5:**
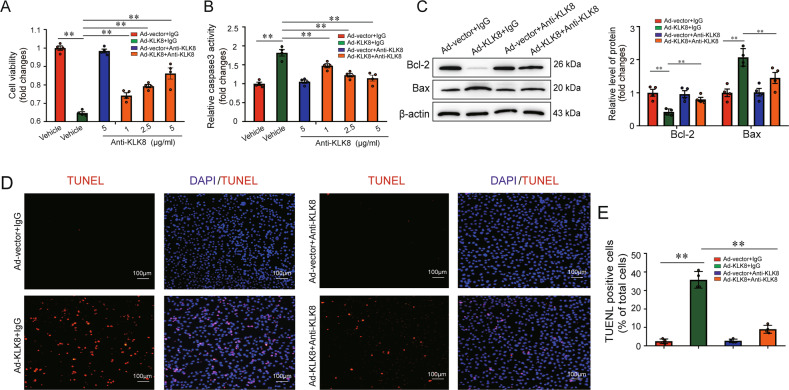
Anti-KLK8 neutralizing antibody rescues KLK8-overexpressed neuron cells from apoptosis. HT22 murine hippocampal neuronal cells were infected with KLK8 adenovirus (Ad-KLK8) at a multiplicity of infection (MOI) of 3 for 24 h with or without anti-KLK8 neutralizing antibody at the indicated doses. **A**, **B** CCK8 assay and measurement of caspase-3 activity showed that anti-KLK8 antibody blocked Ad-KLK8-induced cell injury and caspase-3 activation in a dose-dependent manner in HT22 cells, respectively. **C** Protein levels of Bcl-2 and Bax were determined by western blot analysis. The representative protein bands (left) and the corresponding histograms (right) showed that anti-KLK8 antibody (2.5 μg/ml) reversed Ad-KLK8-induced changes in protein levels of Bcl-2 and Bax. **D** Showed representative TUNEL-stained (red) cells. Nuclei were counterstained with DAPI (blue). Scale bar = 100 μm. **E** The TUNEL-positive cell number (%) was shown as a ratio of the number of TUNEL-positive cells to the total cell number. **D**, **E** Showed that anti-KLK8 antibody (2.5 μg/ml) reversed Ad-KLK8-induced HT22 cell apoptosis. Data were presented as means ± SD (*n* = 4, one-way ANOVA, Bonferroni’s post hoc test). **p < 0.01.

KLK8 is a trypsin-like serine protease [17]. We then used two serine protease inhibitors, Antipain and ZnSO4, to block the proteolytic activity of KLK8, as described previously [26]. As shown in supplemental Fig. 4A, B, both Antipain and ZnSO4 blocked Ad-KLK8-induced cell damage and caspase-3 activity in a dose-dependent manner. In addition, antipain and ZnSO4 increased Bcl-2 expression, whereas decreased Bax expression (supplemental Fig. 4C) and the percentage of TUNEL-positive cells in Ad-KLK8-treated HT22 cells (supplemental Fig. 4D, E). Furthermore, we found that treatment of HT22 cells with increasing concentrations of anti-KLK8 neutralizing antibody dose-dependently attenuated Ad-KLK8-induced increases in HT22 cell damage and caspase-3 activity (Fig. 5A, B). In addition, Ad-KLK8-induced increases in Bax expression (Fig. 5C) and the percentage of TUNEL-positive HT22 cells (Fig. 5D, E) were reduced, while Ad-KLK8-induced decreases in Bcl-2 expression (Fig. 5C) were profoundly prevented by an anti-KLK8 neutralizing antibody. Collectively, these results indicated that the pro-injury and pro-apoptotic effects of KLK8 overexpression were dependent on its proteolytic activity.

**Fig. 6:**
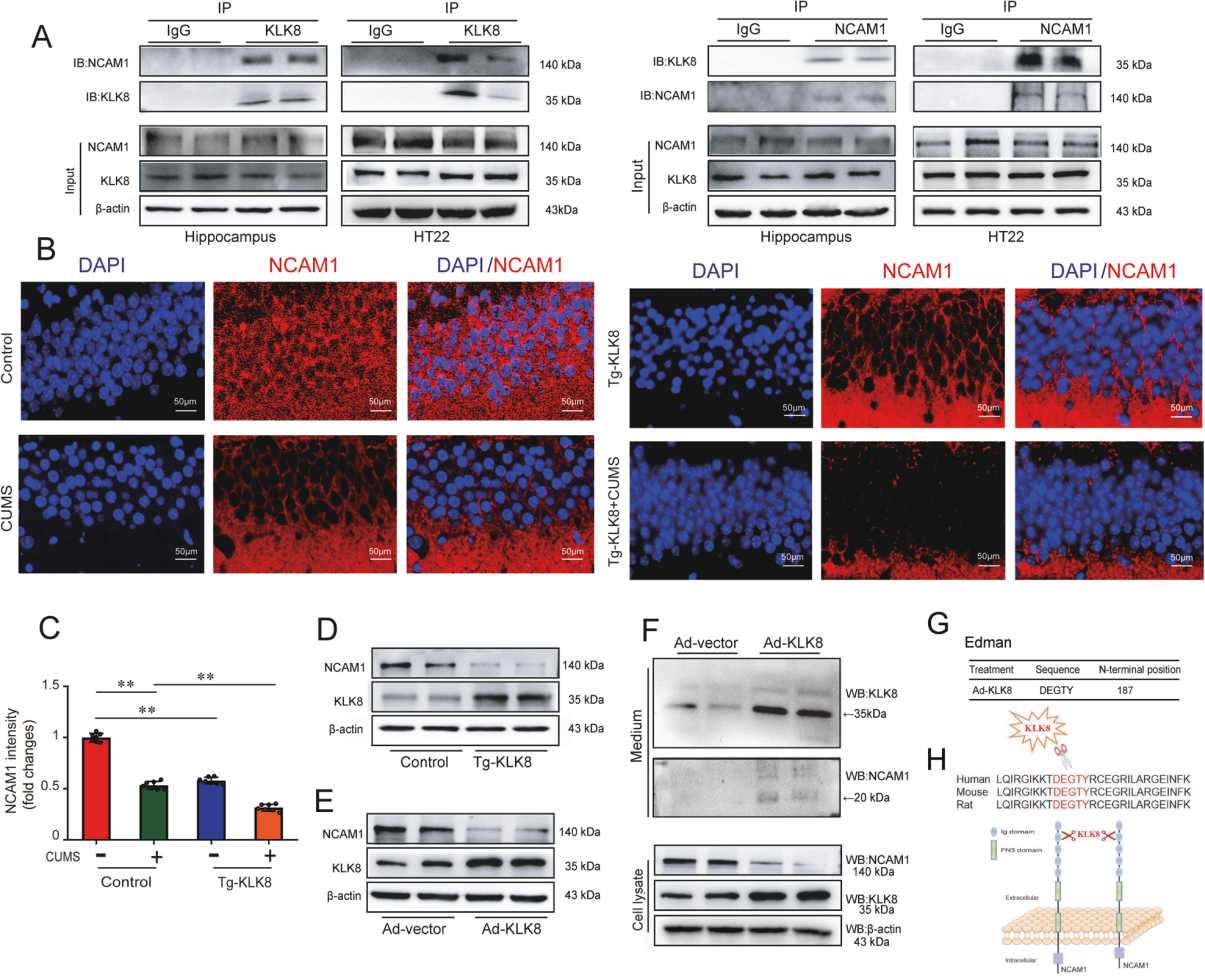
NCAM1 may be recognized as a potential substrate of KLK8 in neurons. **A** Association of NCAM1 and KLK8 was tested by reciprocal immunoprecipitations in rat hippocampus and HT22 cells. IgG was controlled for nonspecific interaction. **B** immunofluorescent staining showed NCAM1 (red) levels in the cryostat-cut hippocampal sections of Tg-KLK8 rats. Nuclei were counterstained with DAPI (blue). Scale bar = 50 μm. **C** Showed quantification of the fluorescence intensity of the NCAM1 (*n* = 7, two-way ANOVA, Bonferroni’s post hoc test). **D**, **E** Protein levels of NCAM1 and KLK8 in the hippocampus of KLK8 transgenic (Tg-KLK8) rats (**D**, *n* = 7, unpaired *t*-test) and KLK8 adenovirus (Ad-KLK8)-treated HT22 cells (**E**, *n* = 4, unpaired *t*-test) were determined by western blot analysis. **F** HT22 cells were treated with Ad-vector or Ad-KLK8 in a serum-free medium for 24 h. Immunoblots showed the appearance of a ~20 kDa N-terminal NCAM1 fragment in the medium. WB western blot. **G** The N-terminal sequence of the ~20 kDa protein band was determined by Edman assay. The sequence, as indicated was, belonging to the NCAM1 sequence. **H** schematic representation of the extracellular part of NCAM1 cleaved by KLK8. NCAM1 extracellular domain consists of five Ig-like modules followed by two fibronectin type III (FnIII) modules. KLK8 cleaved NCAM1 after amino acid 187 in the second Ig-like domain of NCAM1. Data were presented as means ± SD. **p < 0.01.

Using co-immunoprecipitation (Co-IP) combined with mass spectrometry, the present study identified proteins associated with KLK8 in the hippocampus of KLK8 transgenic rats. Mass spectrometry revealed that NCAM1, a key molecule involved in neuronal survival and regeneration [27], was the potentially matched protein (supplemental Table 1). As shown in Fig. 6A, NCAM1 was co-immunoprecipitated by anti-KLK8, and vice versa, in rat hippocampus and HT22 cells. We then observed the expression of NCAM1 in hippocampal sections using immunofluorescent staining. As shown in Fig. 6B, C and supplemental Fig. 5A, B, decreased NCAM1 staining was found in hippocampal sections obtained from mice or rats exposed to CUMS. In KLK8 transgenic rats exposed to CUMS, NCAM1 expression decreased even further in the hippocampal section. In contrast, the CUMS-induced loss of NCAM1 was largely prevented in KLK8-deficient mice. In addition, western blot analysis confirmed that KLK8 overexpression led to the downregulation of NCAM1 in the hippocampus (Fig. 6D and supplemental Fig. 6A) and HT22 cells (Fig. 6E and supplemental Fig. 6B).

To determine whether KLK8 could directly cleave NCAM1, we incubated recombinant NCAM1 with recombinant KLK8 at 37 °C and observed a decrease of NCAM1 in a time-dependent and dose-dependent manner (supplemental Fig. 7A, B). This result indicated that KLK8 directly cleaved NCAM1. The extracellular part of NCAM1 is known to consist of five Ig-like modules followed by two fibronectin type III (FnIII) modules [28]. The present study then examined the composition of the HT22 culture medium after cell transfection with Ad-KLK8. It was found that a new ~20 kDa extracellular fragment of NCAM1 was released into the medium (Fig. 6F). N-terminal sequencing was then performed on this 20-kDa fragment, and the data showed that KLK8 cleaved NCAM1 after amino acid 187 in the second Ig-like domain of NCAM1 (Fig. 6G, H). Collectively, these findings suggested that NCAM1 might be recognized as a potential substrate of KLK8, and the cleaved NCAM1 protein might lack the homophilic binding domain of NCAM1 [29, 30].

### Both NCAM1 overexpression and NCAM1 mimetic peptide rescue KLK8-overexpressed neuron cells from apoptosis

**Fig. 7:**
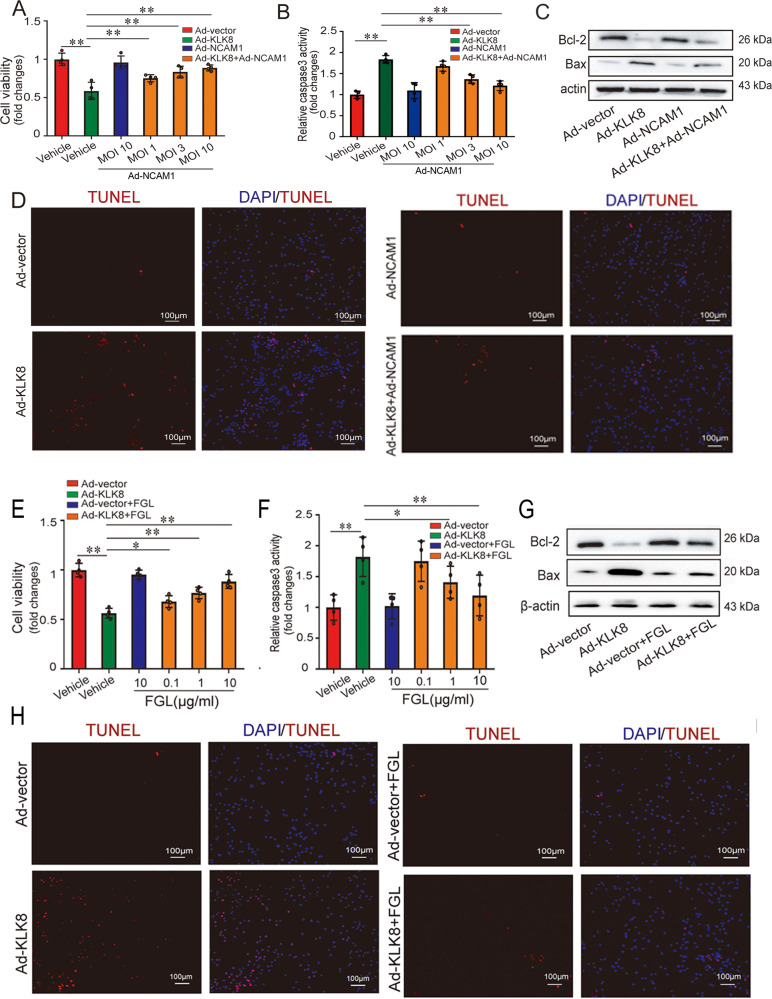
Both NCAM1 overexpression and NCAM1 mimetic peptide FGL rescue KLK8-overexpressed HT22 cells from apoptosis. HT22 murine hippocampal neuronal cells were infected with KLK8 adenovirus (Ad-KLK8) at a multiplicity of infection (MOI) of 3 for 24 h with or without NCAM1 adenovirus (Ad-NCAM1) (**A**–**D**) or NCAM1 mimetic peptide FGL (**E**–**H**). **A**, **B** CCK8 assay and measurement of caspase-3 activity showed that Ad-NCAM1 blocked Ad-KLK8-induced cell injury and caspase-3 activation in a dose-dependent manner in HT22 cells, respectively. **C** Protein levels of Bcl-2 and Bax were determined by western blot analysis. **D** Showed representative TUNEL-stained (red) cells. Nuclei were counterstained with DAPI (blue). Scale bar = 100 μm. **E**, **F** CCK8 assay and measurement of caspase-3 activity showed that FGL peptide blocked Ad-KLK8-induced cell injury and caspase-3 activation in a dose-dependent manner in HT22 cells, respectively. **G** protein levels of Bcl-2 and Bax were determined by western blot analysis. **H** Showed representative TUNEL-stained (red) cells. Nuclei were counterstained with DAPI (blue). Scale bar = 100 μm. Data were presented as means ± SD (*n* = 4, one-way ANOVA, Bonferroni’s post hoc test). *p < 0.05, **p < 0.01.

Both genetic and pharmacologic approaches were then used to further confirm the involvement of NCAM1 in KLK8 overexpression-induced neuronal apoptosis. It was found that NCAM1 adenovirus (Ad-NCAM1) treatment dose-dependently prevented Ad-KLK8-induced increases in HT22 cell damage (Fig. 7A) and caspase-3 activity (Fig. 7B). In addition, NCAM1 overexpression significantly attenuated Ad-KLK8-induced increases in Bax expression (Fig. 7C and supplemental Fig. 8A) and the percentage of TUNEL-positive HT22 cells (Fig. 7D and supplemental Fig. 8B). In contrast, anti-apoptotic protein Bcl-2 expression was increased after Ad-NCAM1 treatment (Fig. 7C and supplemental Fig. 8A).

NCAM1 mimetics are bioactive peptides derived from fragmented NCAM1 sequences and, thus, have a similar mode of action as NCAM1 [28]. They have been successfully used as an alternative for NCAM1 insufficiency. In the present study, we used a synthetic NCAM1 mimetic peptide corresponding to the NCAM1 FnIII2 FG loop (FGL) [28]. As shown in Fig. 7E, F, treatment of HT22 cells with increasing concentrations of FGL peptide dose-dependently attenuated Ad-KLK8-induced increases in HT22 cell damage and caspase-3 activity. In addition, Ad-KLK8-induced increases in Bax expression (Fig. 7G and supplemental Fig. 8C) and the percentage of TUNEL-positive HT22 cells (Fig. 7H and supplemental Fig. 8D) were reduced, while Ad-KLK8-induced decreases in Bcl-2 expression (Fig. 7G and supplemental Fig. 8C) were profoundly prevented by FGL peptide treatment.

**Fig. 8:**
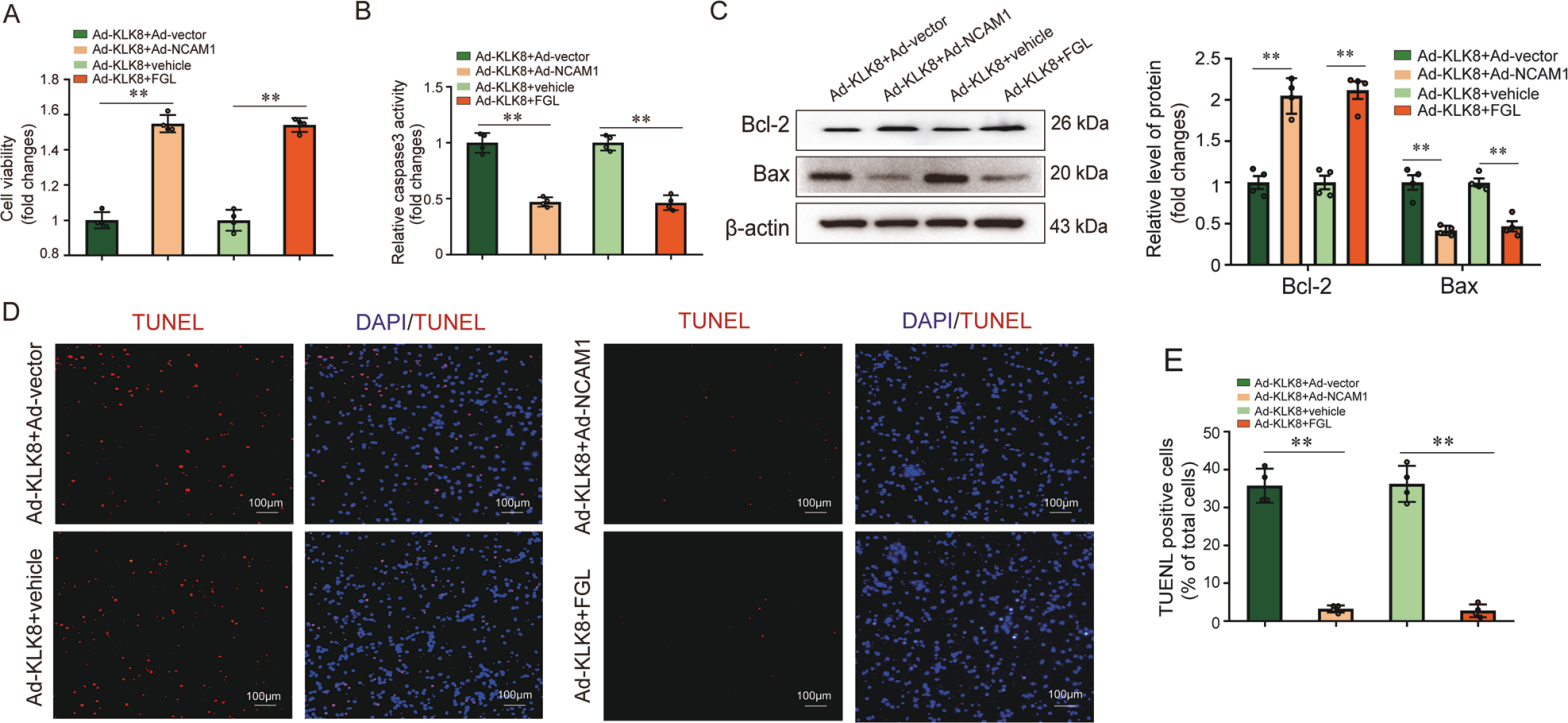
Both NCAM1 overexpression and NCAM1 mimetic peptide FGL rescue KLK8-overexpressed primary hippocampal neurons from apoptosis. Primary hippocampal neurons were infected with KLK8 adenovirus (Ad-KLK8) at a multiplicity of infection (MOI) of 3 with or without NCAM1 adenovirus (Ad-NCAM1, MOI 3) or NCAM1 mimetic peptide FGL (1 μg/ml) for 24 h. **A**, **B** CCK8 assay and measurement of caspase-3 activity showed that both Ad-NCAM1 and FGL blocked Ad-KLK8-induced cell injury and caspase-3 activation in primary hippocampal neurons, respectively. **C** Protein levels of Bcl-2 and Bax were determined by western blot analysis. The representative protein bands (left) and the corresponding histograms (right) showed that both Ad-NCAM1 and FGL peptide reversed Ad-KLK8-induced changes in protein levels of Bcl-2 and Bax. **D** Showed representative TUNEL-stained (red) cells. Nuclei were counterstained with DAPI (blue). Scale bar = 100 μm. **E** The TUNEL-positive cell number (%) was shown as a ratio of the number of TUNEL-positive cells to the total cell number. **D**, **E** Showed that both Ad-NCAM1 and FGL peptide reversed Ad-KLK8-induced primary hippocampal neuron apoptosis. Data were presented as means ± SD (*n* = 4, one-way ANOVA, Bonferroni’s post hoc test). **p < 0.01.

The effect of NCAM1 overexpression and NCAM1 mimetics on KLK8 overexpression-induced cell apoptosis was also observed in primarily isolated hippocampal neurons. It was found that both NCAM1 overexpression and FGL peptide treatment prevented Ad-KLK8-induced increases in cell damage (Fig. 8A) and caspase-3 activity (Fig. 8B). In addition, NCAM1 overexpression and FGL peptide treatment significantly attenuated Ad-KLK8-induced increases in Bax expression (Fig. 8C) and the percentage of TUNEL-positive cells (Fig. 8D, E). In contrast, anti-apoptotic protein Bcl-2 expression was increased by treatment with Ad-NCAM1 or FGL peptide in primary hippocampal neurons (Fig. 8C). Collectively, these results indicated that both NCAM1 overexpression and NCAM1 mimetic peptide rescued KLK8-overexpressed neuron cells from apoptosis.

## Discussion

KLK8 (also known as neuropsin) is highly expressed in the hippocampus, amygdala, and other brain regions associated with emotion management, and plays a key role in neuroplasticity, learning, and memory processes [17, 18, 31, 32]. Previous studies have suggested the relationship between KLK8 and behavioral regulation. For example, peripheral blood KLK8 mRNA levels are significantly higher in patients with the major recurrent depressive disorder compared to healthy subjects [33]. Hippocampal KLK8 mRNA expression is increased after both acute restraint stress and chronic stress [21, 34]. Antidepressants are found to alter the expression of KLK8 in the hippocampus [35]. Several studies have suggested the critical role of KLK8 in the pathogenesis of anxiety-related behaviors due to different etiologies [21, 36–38]. However, to the best of our knowledge, only one study reported that the severity of depression-like behaviors induced by chronic stress or chronic corticosterone injection is reduced in KLK8-deficient mice [31]. The present study reported for the first time that increased KLK8 protein levels in the hippocampus was associated with CUMS-induced depression-like behaviors. KLK8 deficiency significantly alleviated, whereas transgenic overexpression of KLK8 exacerbated CUMS-induced depression-like behaviors. These findings suggest that the upregulation of KLK8 may contribute to the pathogenesis of CUMS-induced depression.

An important implication of hippocampal KLK8 upregulation contributing to CUMS-induced depression-like behavior is that KLK8-targeted therapy may have clinical relevance for patients with depression. In fact, increased levels of KLK8 have been implicated in the pathogenesis of various brain diseases, including schizophrenia, mood and anxiety disorders, autoimmune encephalomyelitis, and Alzheimer’s disease [17, 37–39]. The critical role of KLK8 in this wide spectrum of diseases motivated the development of KLK8-targeted therapies such as macroglobulins, peptide-based and small molecule inhibitors, and antibodies [39, 40]. Notably, two preclinical studies have demonstrated that inhibition of excessive cerebral KLK8 by intraventricular anti-KLK8 antibody delivery can enhance neuroplasticity, reverse molecular signatures of anxiety, and ultimately improve memory and reduce fear in Alzheimer’s disease mouse model [36, 39]. On the other hand, KLK8 reduction beneath or rise above physiological levels both impairs spatial memory performance in healthy wild-type mice. Anti-KLK8 antibody administration elicits anxiolytic effects and exhibits increased exploratory behavior in wild-type mice [36]. Taken together, although these findings prompted us to consider antibody-mediated KLK8 inhibition as a promising therapeutic strategy against depression, the anti-KLK8 antibody must be used with great caution due to its side effects.

Apoptosis plays an important role in the functional organization of the nervous system by controlling the number of neurons [41]. Accumulating laboratory studies have recognized that neuronal apoptosis in the hippocampus is one of the major causes of depression-like phenotypes [14–16]. The present study demonstrated that KLK8 deficiency significantly alleviated, whereas transgenic overexpression of KLK8 exacerbated CUMS-induced hippocampal neuron apoptosis. In vitro studies demonstrated that KLK8 overexpression directly induced cell apoptosis in hippocampal neuronal cells. Collectively, our findings suggest that the upregulation of KLK8 may contribute to the pathogenesis of CUMS-induced depression by promoting neuronal apoptosis in the hippocampus.

KLK8 is a secreted serine protease that exhibits proteolytic activity with a trypsin-like substrate specificity [17, 18]. KLK8 plays an important role in the breakdown of extracellular matrix proteins such as fibronectin [42]. KLK8 is also known to cleave the extracellular portion of several membrane proteins, including synaptic adhesion molecule L1 [43], neuregulin-1 [44], and ephrin type-B receptor 2 [21], which has been implicated in the pathogenesis of psychiatric disorders. In the present study, multiple-line evidence revealed that KLK8 was critically involved in the proteolytic processing of NCAM1, a major neural adhesive protein closely associated with neuronal survival and neurogenesis in the hippocampus.

Ectodomain shedding of NCAM1 in the various types of neurons is of critical importance for neuronal survival, synaptic plasticity, cognitive function, and behavioral development [45–47]. Members of the matrix metalloproteinases (MMPs) and disintegrin and metalloproteinases (ADAMs) have been found to mediate the proteolytic degradation of NCAM1, thus impairing neuronal connectivity, synaptic plasticity and promoting microglial activation [48–51]. In particular, MMP9-mediated proteolytic degradation of NCAM1 is involved in ischemic stress-induced neuronal damage [47] and oxidative stress-induced neuronal death [52]. The present study additionally contributed to the understanding of this complex system, demonstrating that KLK8 mediated the ectodomain shedding of NCAM1, which may represent a potent mechanism for neuronal apoptosis in the hippocampus during the pathogenesis of CUMS-induced depression.

Dysregulated NCAM1 has been found in patients with major depression [53]. Mice with reduced or deficient NCAM exhibit depression-like behaviors, which can be reversed by the administration of FGL peptide [54, 55]. Inactivation of the NCAM gene in the forebrain display increased vulnerability to stress-induced depression-like behaviors [56]. In the present study, we found that KLK8 overexpression reduced NCAM1 expression, while KLK8 deficiency reversed CUMS-induced downregulation of NCAM1 in the hippocampus. Moreover, both NCAM1 overexpression and NCAM1 mimetic peptide rescued KLK8-overexpressed neuron cells from apoptosis. These findings suggest that the antidepressant effects of KLK8 deficiency may be attributed at least partly to the restoration of hippocampal NCAM1 levels in CUMS-exposed mice. Notably, previous studies have demonstrated the significant abnormality in the hippocampal neural system of KLK8-deficient mice, including synaptic loss, an increase of fast-spiking interneurons, and increased susceptibility for hyperexcitability in response to repetitive afferent stimulation [57, 58]. Therefore, it cannot be excluded that additional mechanisms, such as synaptic, morphological, and electrophysiological disturbances, may also contribute to the improvement of depression symptoms observed in KLK8-deficient mice exposed to CUMS.

Previous studies have reported that the synthetic NCAM-ligand or NCAM mimic may inhibit cell apoptosis in cerebellar and dopaminergic neurons by stimulating PI3K/Akt signaling pathway [59, 60]. Recently, NCAM is also found to exert anti-apoptotic effects by modulating the activity of glycogen synthase kinase 3β (GSK3β) in dorsal root ganglion and spinal cord motor neurons, as well as in sympathetic neuron-like PC12 cells [61, 62]. In light of these findings, studies investigating the contribution of PI3K/Akt, GSK3β, and other potential downstream signaling molecules to NCAM1-dependent neuroprotective effects in the hippocampus will be of considerable interest.

There are three main limitations in this study. First, KLK8 transgenic rats and KLK8 knockout mice were used to assess the impact of KLK8 overexpression and KLK8 deficiency on CUMS-induced depression, respectively. Interpretations of results from opposite manipulations performed in different species must be made with caution. Second, previous studies have shown a sexually dimorphic expression of KLK8 in both neonatal and adult hippocampus [63, 64]. Estrogen, but not testosterone, induces KLK8 expression in neuronal and microglial cell lines [63]. Thus, the present study only used male rats and mice to exclude the potential interference of estrogen on hippocampal KLK8 expression. Notably, Keyvani et al. reports that higher levels of KLK8 in the female brain may increase the risk for Alzheimer’s disease [63]. These findings suggest that future investigations should explore the potential role of KLK8 in sex differences of depressive disorders. Third, transient overexpression of KLK8 and NCAM1 was used to assess the effects of KLK8 and NCAM1 on hippocampal neuron apoptosis in vitro. Given that transient overexpression can violate balanced gene dosage and affect protein folding and assembly [65], caution should be made in interpreting cell apoptosis data based on transient overexpression experiments.

In summary, the present study provided several lines of evidence supporting a new pro-apoptotic mechanism in the hippocampus during the pathogenesis of CUMS-induced depression via the upregulation of KLK8. KLK8 mediated the proteolytic processing of the NCAM1 extracellular domain, thus exerting a pro-apoptotic effect on hippocampal neurons. Our present data raised the possibility of KLK8 as a potential therapeutic target for depression and provided a new mechanism for CUMS-induced depression.

## Supplementary information

supplementary files clean

suppleement material

Checklist

## Data Availability

All data generated or analyzed during this study are available from the corresponding author on reasonable request.
