# Supplementary material for: Upregulation of KLK8 contributes to CUMS-induced hippocampal neuronal apoptosis by cleaving NCAM1
Source: Cell Death Dis. 2023 Apr 19;14(4):278. doi: 10.1038/s41419-023-05800-5 (PMC10115824; doi:10.1038/s41419-023-05800-5)
Supplement: Supplementary file 1 — supplementary files clean [file 41419_2023_5800_MOESM1_ESM.doc]

Supplemental Digital Content

**Upregulation of KLK8 contributes to CUMS-induced hippocampal neuronal apoptosis by cleaving NCAM1**

Dan-Hong Xu^1,2a^, Jian-Kui Du^3a^, Shi-Yu Liu^1a^, Hui Zhang^4^, Lu Yang^1^, Xiao-Yan Zhu^2*^, Yu-Jian Liu^1*^

1-School of Kinesiology, Shanghai Frontiers Science Research Base of Exercise and Metabolic Health, The Key Laboratory of Exercise and Health Sciences of Ministry of Education Shanghai University of Sport, Shanghai, 200438.

2- Department of Physiology, Navy Medical University, Shanghai, 200433.

3- National Clinical Research Center for Geriatric Disorders and National International Joint Research Center for Medical Metabolomics, Xiangya Hospital, Central South University, Changsha, Hunan, China, 41008.

4-Department of Anesthesiology and Surgical Intensive Care Unit, Xinhua Hospital, Shanghai Jiaotong University School of Medicine, Shanghai, 200092.

a These authors contributed equally to this work and should be considered as co-first authors

*** Corresponding Authors:**

Prof. Yu-Jian Liu: School of Kinesiology, The Key Laboratory of Exercise and Health Sciences of Ministry of Education Shanghai University of Sport, 200 Hengren Road, Shanghai 200438, China. Email: liuyujian@sus.edu.cn;

Prof. Xiao-Yan Zhu: Department of Physiology, Navy Medical University, Shanghai, 200433. China. Email: [xiaoyanzhu@smmu.edu.cn](mailto:xiaoyanzhu@smmu.edu.cn)

**Materials and Methods**

**Animals**

All laboratory mice and rats in this study were maintained in a pathogen-free facility at the Animal Research Center of Navy Medical University. Animal studies were performed in accordance with the Guide for the Care and Use of Laboratory Animals published by the NIH (NIH publication No. 85-23, revised 1996), and were approved by the Ethical Committee of Experimental Animals of Shanghai University of Sport. Animal experiments were randomized with a random number table. All in vivo studies were blinded for both genotype and treatment during the measurement and analysis stages. To exclude the potential interference of different sexes, only male mice and rats were included in the experiments of this study.

The present study used global KLK8 knockout mice and KLK8 transgenic rats generated by our research group, and the identification method was the same as previously described by our research group [S1, 2]. The KLK8-flox mouse line was generated at the Shanghai Biomodel Organism Science & Technology Development Co., Ltd (Shanghai, China) using a LoxP targeting system with two LoxP elements flanking exon 1-3 of KLK8. Briefly, the two LoxP elements were inserted into the KLK8 gene by homologous recombination in embryonic stem (ES) cells. To generate global KLK8 knockout mice, KLK8-flox mice were mated with EIIa-Cre transgenic mice (The Jackson Laboratory). Deletion of the KLK8 gene was verified by PCR of genomic DNA using (5’-GGACGTTGGAGTCACAGC-3’) and (5’-CCCAGGAGCAGAAGAGTG-3’) primers. KLK8^flox/flox^; EIIa–Cre(+) mice (KLK8-/-), along with age-matched KLK8^flox/flox^; EIIa-Cre (-) littermates as controls, were used to investigate the impacts of KLK8 deficiency.

F0 transgenic Sprague-Dawley rat embryos were generated by pronuclear injection of a CMV promoter-controlled expression vector containing the rat KLK8 cDNA by Cyagen Biosciences Inc. (Guangzhou, China). Genotyping of KLK8 transgenic rats was determined by PCR screening of genomic DNA extracted from rat-tail biopsies using two primer pairs called CMV-F/KLK8-R and KLK8-F/IRES-R. The primer sequences are as follows: CMV-F: CATCGCTATTACCATGGTGATG, KLK8-R: AGGCTGTCTGCCAAGGTTG. Animals with the same genotype were assigned to 1:1 allocation ratio to a normal group and a CUMS model group.

**Chronic unpredictable mild stress (CUMS)**

The animals were subjected to chronic unpredictable mild stress (CUMS) as previously described [S3]. Briefly, the CUMS procedure used in this study involved the following 9 mild stressors: continuous overnight illumination, cage tilting, water deprivation, food deprivation, shaker stress, soiled cage, physical restraint, hot stress in oven, forced swimming. The stressors were randomly performed every day for 5 weeks, but the same stressor was never applied on two consecutive days. The animals in the control group were housed separately in another room to avoid the influence of the stress stimulation. Behavioral measurements were performed after 5 weeks of CUMS.

**Behavioral measurements**

Sucrose Preference Test (SPT) was performed as described previously [S4]. Briefly, the rats and mice were first habituated to drink 1% sucrose solution from two bottles for 24 h. After fasting and water deprivation for the following 24 h, the animals were allowed to drink one bottle of 1% sucrose solution and another bottle of water freely for 3 h. The water and sucrose consumption were measured, and the preference for sucrose over water [sucrose solution / (distilled water + sucrose solution) * 100 %] was used as a measure of the reduced ability to experience pleasure.

The novelty-suppressed feeding test (NSFT) was performed as described previously [S5]. Animals were deprived of food 24 h prior to the test. In the test, equal amounts of food were placed in the center of the apparatus (rats: 40 cm length×40 cm width×40 cm height; mice: 25cm length×25 cm width×30 cm height). The animals were placed in the corner of the apparatus, biting the food freely for 10 minutes, and the time to the first feeding was recorded. Immediately afterward, the animals were returned to the cages, and food and water were provided ad libitum.

Forced swimming test (FST) was performed as the method described previously [S6] with minor modifications. Animals were placed in Plexiglas cylinders (rats: 30 cm diameter×80 cm high, mice: 20 cm diameter×35 cm high) filled with water at 23-25°C. All animals were forced to swim for 6 min, and resting time and swimming time were recorded during the last 5 min of the experiment.

Tail suspension test (TST) was conducted as described previously [S7]. Each mouse was fixed with tape approximately 1 cm from the tip of the tail for 6 min, and the duration of immobility during the last 5 min of the test was recorded.

**Cell culture and adenoviral infection**

HT22 murine hippocampal neuronal cells (ATCC, Rockville, MD, USA) were maintained in DMEM (HyClone, Utah, USA) supplemented with 10 % FBS (Gibco, California, USA) in a humidified 5 % CO2 incubator at 37 °C. Because of the high metabolic needs, optimal growth and survival rate of HT22 cells require 25 mM basal glucose. Hence, high glucose DMEM medium (25 mM glucose) was used to meet these metabolic requirements.

KLK8 adenovirus was generated by using the AdEasyTM adenoviral vector system (Stratagene, La Jolla, CA, USA) as previously described [1]. The cell infection was performed according to the manufacturer’s protocol in high glucose DMEM medium supplemented with 10 % FBS. Serum-free medium was only used in the experiment examining the composition of the HT22 culture medium after cell transfection with Ad-KLK8, in order to exclude the interference of serum proteins.

**Hippocampal primary neuron culture**

Primary hippocampal neurons were prepared from postnatal (days 1-3 after birth) mice as described previously [S8]. The hippocampus was minced and digested with 0.25% trypsin for 15 min at 37 °C. Neurons were grown in Neurobasal medium (Gibco, CA, USA) supplemented with B-27 (Invitrogen, 1:50), GlutaMax-I (Invitrogen, 1:100), and 100 μg/ml penicillin/streptomycin at 37 °C in a humidified 5 % CO2 incubator at 37 °C.

**Cell viability assay**

HT22 cells seeded in 96-well plates (100 μL/well) were infected with KLK8 adenovirus for 24 h. Cell viability was assayed using Cell Counting Kit-8 (CCK8, Beyotime, Jiangsu, China) according to the manufacturer's instructions. The absorbance of each well was measured, and the OD at 490 nm was obtained using an enzyme-labeled instrument.

**Measurement of caspase-3 activity**

Caspase-3 activity in the HT22 cells was measured by using the caspase-3 activity assay kit (Beyotime, Jiangsu, China) as previously described [S7]. Caspase-3 activity assay kit is based on the fact that caspase-3 can catalyze the production of yellow pNA (p-nitroaniline) from the substrate Ac-DEVD-pNA (acetyl-Asp-Glu-Val-Asp p-nitroanilide), thus allowing the detection of caspase-3 by measuring the absorbance activity. In brief, HT22 was homogenized in lysis buffer and centrifuged at 20000 rpm for 15 minutes at 4°C, followed by absorbance at 405 nm to quantify caspase-3 activity.

**TdT-mediated dUTP nick-end labeling (TUNEL) assay**

Briefly, hippocampal cryosections (20μm) and cultured HT22 cells were fixed in 4 % paraformaldehyde for 30 min at room temperature, followed by permeabilization in 0.1 % sodium citrate containing 0.5 % Triton X-100 for 5 min as described previously [9], and the hippocampal cryosections and HT22 cells were stained using the One Step TUNEL Apoptosis Assay Kit (Beyotime, Jiangsu, China). DAPI staining was used to determine the number of cell nuclei. TUNEL signals were observed with a fluorescence microscope (Olympus, Japan). The percentage of TUNEL-positive cells was determined as the ratio of the number of TUNEL-positive nuclei to the number of DAPI-positive nuclei. The results of the relative fluorescence intensity of TUNEL were estimated by Image Pro Plus software.

Immunofluorescence

hippocampal cryosections (20 μm) were blocked with 5% goat serum for 2 h at room temperature, then incubated overnight at 4 °C with primary antibodies against NCAM1 (Proteintech, 14255-1-AP). After washes, sections were incubated with secondary antibodies conjugated with Alexa Fluor 568 (Invitrogen, Carlsbad, CA) at 37°C for 1 h in the dark. The sections were counterstained with DAPI for 10 min at room temperature. The fluorescent images were captured by Pannoramic MIDI (3D HISTECH, Budapest, Hungary) and analyzed using Image J software.

**Western blot and Immunoprecipitation**

Hippocampus tissue or HT22 cells using RIPA (Beyotime, Jiangsu, China) containing protease and phosphatase inhibitor cocktail (Beyotime, Jiangsu, China) according to the manufacturer’s instructions. Protein concentration was measured by BCA Protein Assay Kit (Beyotime, Jiangsu, China). Equal amounts of protein were separated with 10 % SDS-PAGE, transferred to PVDF membrane (Millipore, Billerica, MA, USA). The membranes were blocked with 5 % nonfat milk, then incubated with primary antibodies against KLK8 (Abcam, ab232839), NCAM1 (Proteintech, 14255-1-AP), Bax (Servicebio, GB113375), Bcl-2 (Abcam, ab182858), β-actin (Sigma-Aldrich, Mo, USA). Then, the membrane was incubated with a secondary horseradish peroxidase-conjugated antibody for 1 h at room temperature. Immunoreactive proteins were visualized using the chemiluminescence imaging system (Tanon, Shanghai, China).

For Immunoprecipitation assay, hippocampus tissue or HT22 cell lysates were incubated on ice for 2 h, and cell debris was removed by centrifugation. The clarified supernatants were incubated with antibodies against KLK8 (Santa Cruz, sc-67666), NCAM1 (Proteintech, 14255-1-AP) at 4 °C for 16 h with gentle rotation. IgG was used as control for nonspecific interaction. Protein A and G sepharose beads (Beyotime, Jiangsu, China) were added and incubated at 4 °C for an additional 3 h, and the immune complexes were washed four times. The final precipitate was boiled in a protein loading buffer for 5 min and eluted on 10 % SDS-PAGE for western blot analysis using the respective antibodies.

**Mass Spectrometry**

Mass Spectrometry and Bioinformatics was performed as described previously [4]. Proteins were extracted from hippocampal tissue of KLK8 transgenic rats and immunoprecipitated with primary antibodies against KLK8. The immunoprecipitates were separated by SDS-PAGE and stained with the Colloidal Blue Staining kit (Beyotime, Jiangsu, China). Protein sections from the SDS-PAGE gel were digested in the gel with trypsin to extract the peptide markers and the resulting peptide mixture was resuspended in 1% formic acid and identified by ultra performance liquid chromatography tandem mass spectrometry (UPLC-MS/MS) (Bioclouds, Shanghai, China). Briefly, peptide samples were separated with the nano ACQUITY UPLC (Waters Corporation, Milford) and detected with the Q Exactive hybrid quadrupole-Orbitrap mass spectrometer (Thermo Fisher Scientific). The MS/MS spectra were preprocessed with PEAKS studio version 8.5 (Bioinfor Inc., CA) and the PEAKS DB was searched against the Rattus database (UniProtKB/Swiss-Prot). The following search parameters were used: Fixed modifications: Carbamidomethyl (C); Acetylation (Protein N-term), Deamidation (NQ), Variable modifications: Oxidation (M); Missed cleavages: 2; MS mass tolerance: ± 10.0 ppm; MSMS mass tolerance: ± 0.02 Da.

**N-terminal protein sequencing**

HT22 cells were exposed to KLK8 adenovirus in serum-free medium for 24 hours. Supernatants were collected and then concentrated by ultrafiltration. Samples were then separated by SDS-PAGE and transferred to PVDF membranes. The membranes were stained with Coomassie blue staining solution. Bands of ~20 kDa were cut from the membranes and collected. N-terminal sequencing was then performed on a PPSQ-33A system (Kyoto, Japan) at Biotech Pack Scientific (Beijing, China) using the Edman degradation method [4].

**Statistical analysis**

All data are expressed as means ± SD. Two-tailed unpaired t-tests was used to compare the differences between the means of two groups. One-way or two-way analysis of variance (ANOVA) with Bonferroni’s post hoc test was performed for comparisons among multiple groups using SPSS 22.0 (SPSS Inc., Chicago, USA). The correlation analyses were performed using Pearson correlation. P < 0.05 was considered statistically significant.

**References**

S1. Du JK, Yu Q, Liu YJ, Du SF, Huang LY, Xu DH, et al. A novel role of kallikrein-related peptidase 8 in the pathogenesis of diabetic cardiac fibrosis. Theranostics. 2021; 11**:** 4207-4231.

S2. Cao B, Yu Q, Zhao W, Tang Z, Cong B, Du J, et al. Kallikrein-related peptidase 8 is expressed in myocardium and induces cardiac hypertrophy. Sci Rep. 2016; 7**:** 20024.

S3. Hu C, Luo Y, Wang H, Kuang S, Liang G, Yang Y, et al. Re-evaluation of the interrelationships among the behavioral tests in rats exposed to chronic unpredictable mild stress. PLoS One. 2017; 12**:** e0185129.

S4. Wan YQ, Feng JG, Li M, Wang MZ, Liu L, Liu X, et al. Prefrontal cortex miR-29b-3p plays a key role in the antidepressant-like effect of ketamine in rats. Exp Mol Med. 2018; 50**:** 1-14.

S5. Stedenfeld KA, Clinton SM, Kerman IA, Akil H, Watson SJ, Sved AF. Novelty-seeking behavior predicts vulnerability in a rodent model of depression. Physiol Behav. 2011; 103**:** 210-216.

S6. Shang X, Shang Y, Fu J, Zhang T. Nicotine Significantly Improves Chronic Stress-Induced Impairments of Cognition and Synaptic Plasticity in Mice. Mol Neurobiol. 2017; 54**:** 4644-4658.

S7. Castagné V, Moser P, Roux S, Porsolt RD. Rodent models of depression: forced swim and tail suspension behavioral despair tests in rats and mice. Curr Protoc Neurosci. 2011; Chapter 8**:** Unit 8.10A.

S8. Kim H, Kim B, Kim HS, Cho JY. Nicotinamide attenuates the decrease in dendritic spine density in hippocampal primary neurons from 5xFAD mice, an Alzheimer's disease animal model. Mol Brain. 2020; 13**:** 17.

S9. Wang C, Du J, Du S, Liu Y, Li D, Zhu X, et al. Endogenous H(2)S resists mitochondria-mediated apoptosis in the adrenal glands via ATP5A1 S-sulfhydration in male mice. Mol Cell Endocrinol. 2018; 474**:** 65-73.

**Supplemental Table 1. Mass spectrometry analysis of the proteins pulled down by KLK8.**


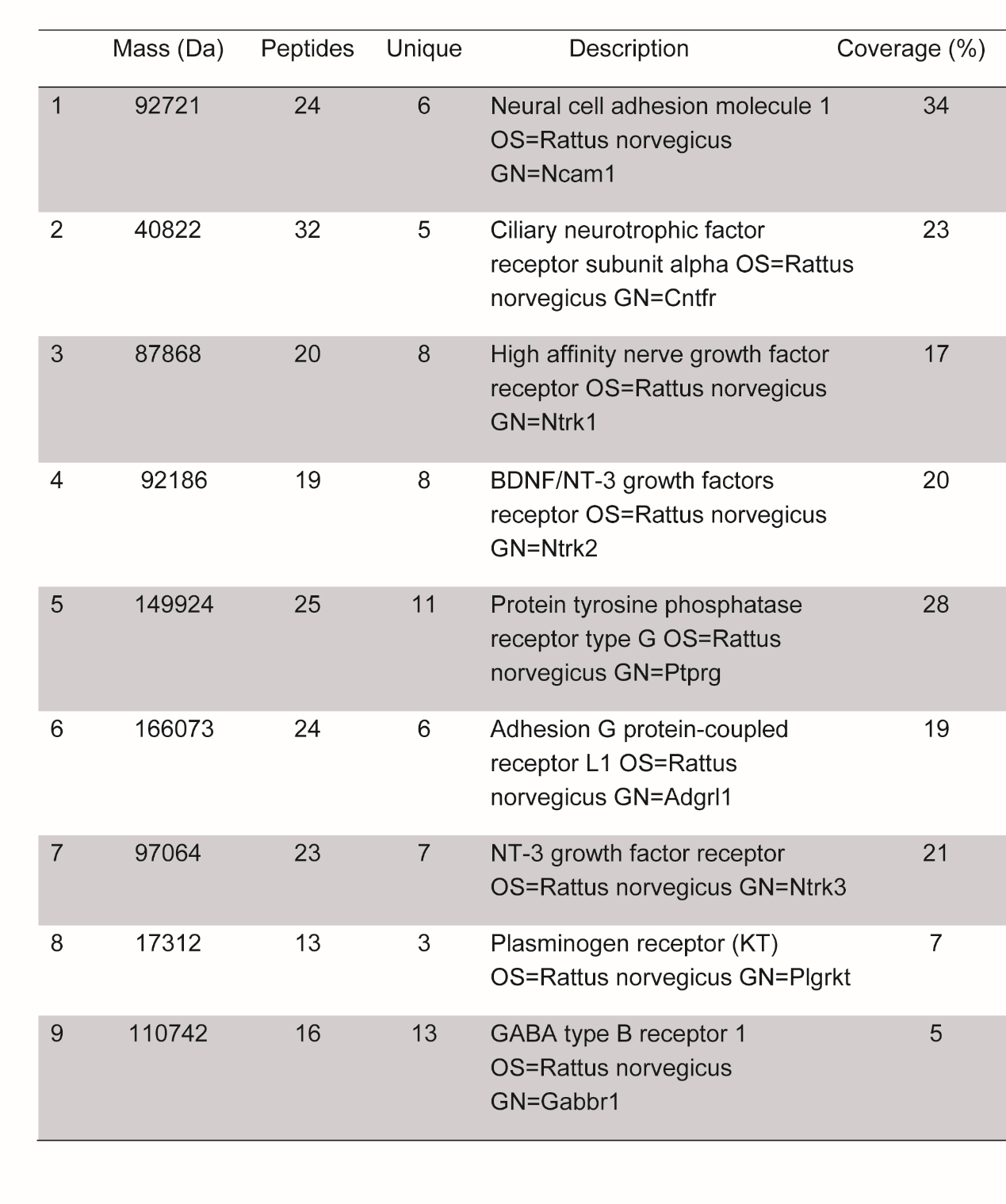


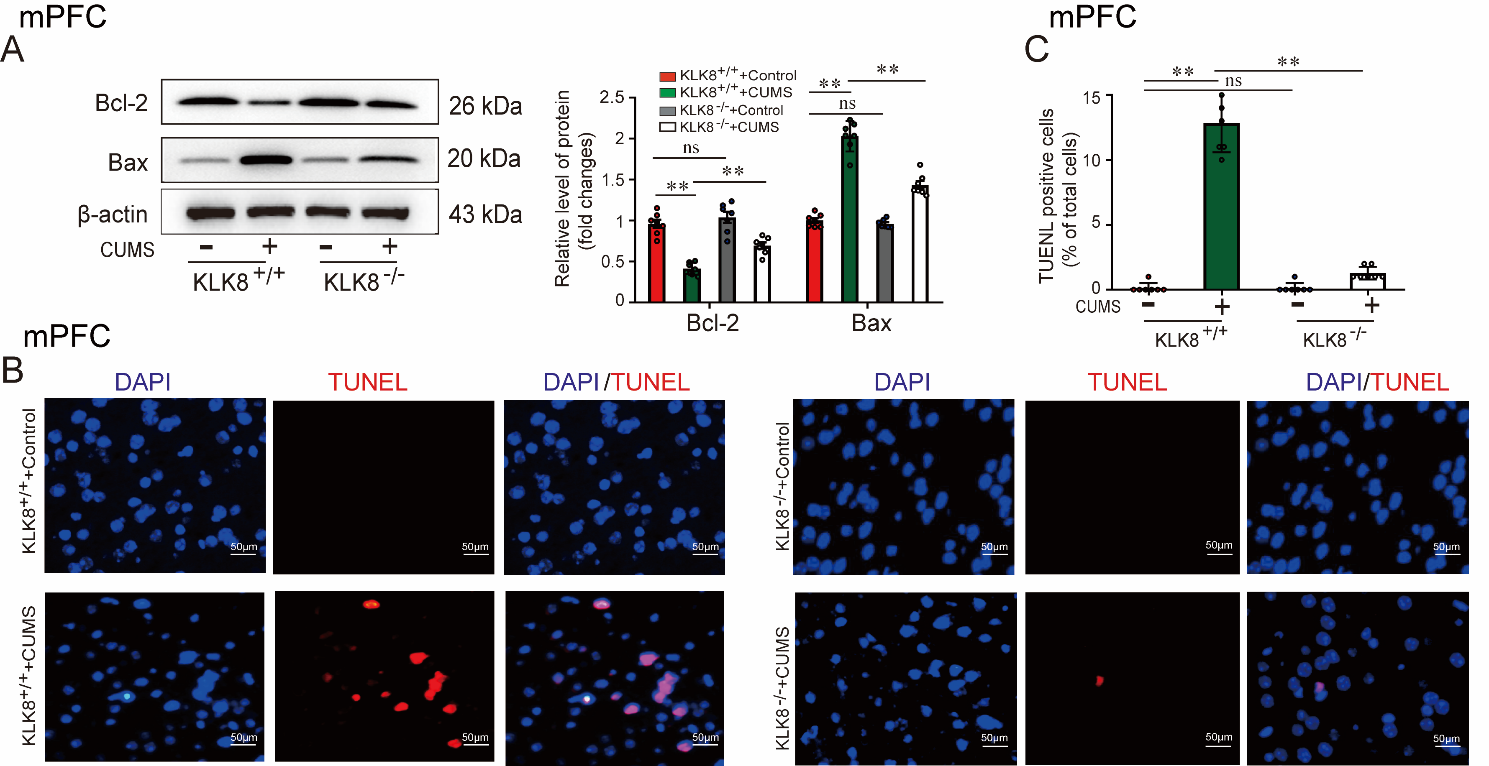


**Supplemental Fig. S1 KLK8 deficiency attenuates CUMS-induced apoptosis in medial prefrontal cortex.** KLK8 deficient (KLK8-/-) mice were exposed to CUMS for 5 weeks. A, protein levels of Bcl-2 and Bax in medial prefrontal cortex (mPFC) of KLK8-/- mice were determined by western blot analysis. Representative protein bands were presented on the left of the histograms. B, showed representative TUNEL-stained cells (red) in the cryostat-cut mPFC sections of KLK8-/- mice. Nuclei were counterstained with DAPI (blue). Scale bar = 50 μm. C, the TUNEL-positive cell number (%) was shown as a ratio of the number of TUNEL-positive cells to the total cell number. Data were presented as means ± SD (n = 7, two-way ANOVA, Bonferroni’s post hoc test). ** p < 0.01, ns, not significant.


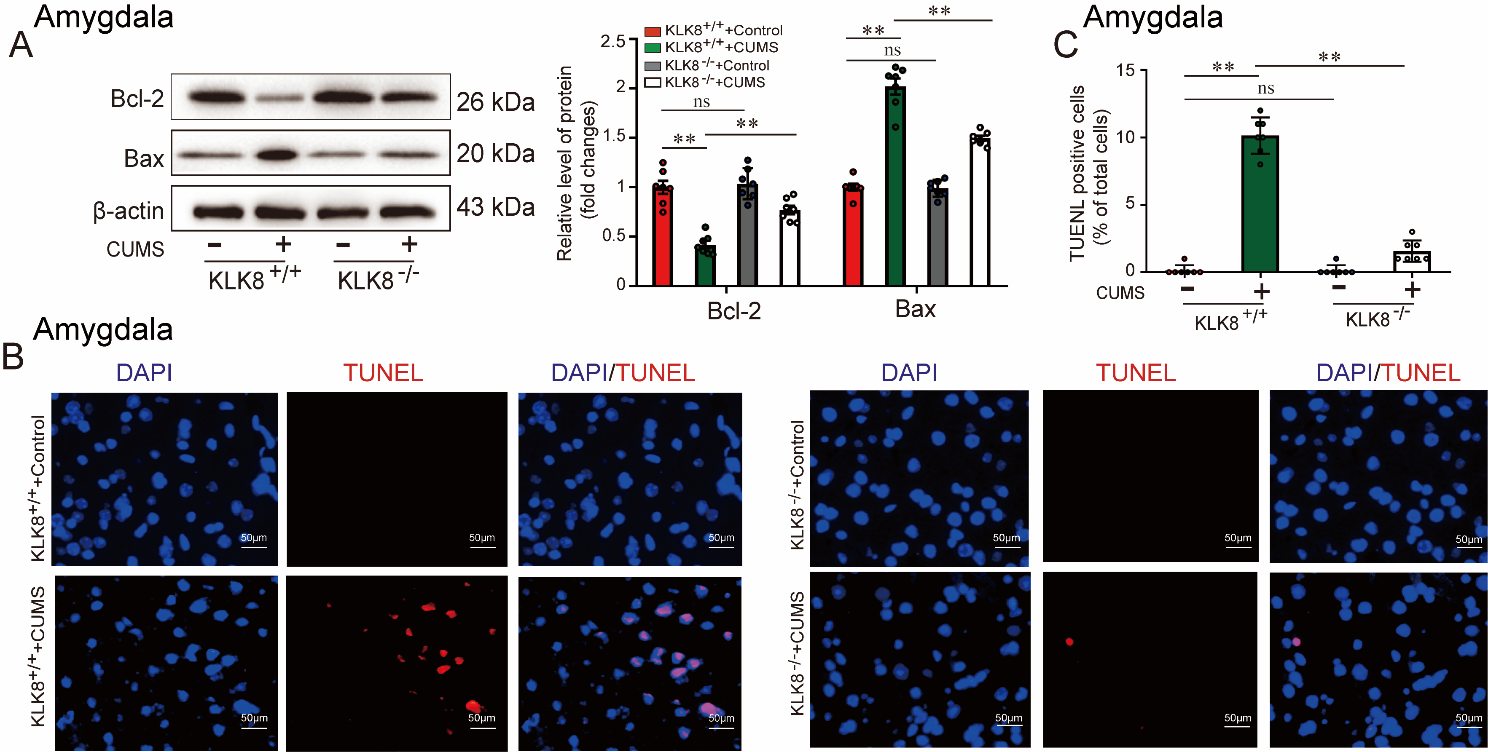


**Supplemental Fig. S2 KLK8 deficiency attenuates CUMS-induced apoptosis in amygdala.** KLK8 deficient (KLK8-/-) mice were exposed to CUMS for 5 weeks. A, protein levels of Bcl-2 and Bax in amygdala of KLK8-/- mice were determined by western blot analysis. Representative protein bands were presented on the left of the histograms. B, showed representative TUNEL-stained cells (red) in the cryostat-cut amygdala sections of KLK8-/- mice. Nuclei were counterstained with DAPI (blue). Scale bar = 50 μm. C, the TUNEL-positive cell number (%) of KLK8-/- mice was shown as a ratio of the number of TUNEL-positive cells to the total cell number. Data were presented as means ± SD (n = 7, two-way ANOVA, Bonferroni’s post hoc test). ** p < 0.01, ns, not significant.


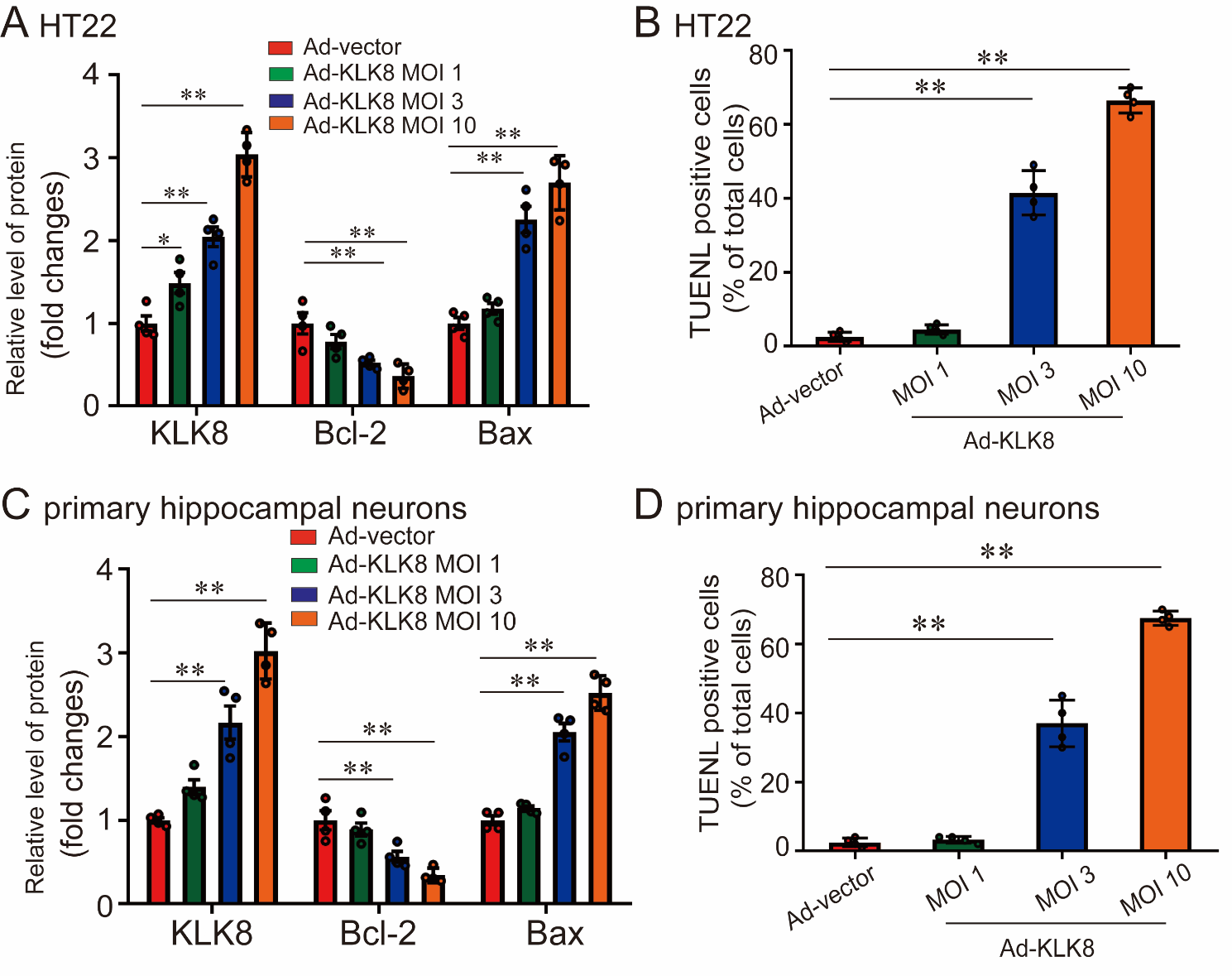


**Supplemental Fig. S3 KLK8 overexpression induces neuron apoptosis *in vitro* (related to Fig. 4).** HT22 murine hippocampal neuronal cells (A-B) and primary hippocampal neurons (C-D) were infected with KLK8 adenovirus (Ad-KLK8) at a multiplicity of infection (MOI) of 1, 3, or 10 for 24 h. A and C, showed relative densitometry of the KLK8, Bcl-2 and Bax protein band in HT22 cells (A) and primary hippocampal neurons (C), respectively. B and D, the TUNEL-positive cell number (%) in HT22 cells (B) and in primary hippocampal neurons (D) was shown as a ratio of the number of TUNEL-positive cells to the total cell number. Data are expressed as means ± SD (n = 4, one-way ANOVA, Bonferroni’s post hoc test). * p < 0.05, ** p < 0.01.


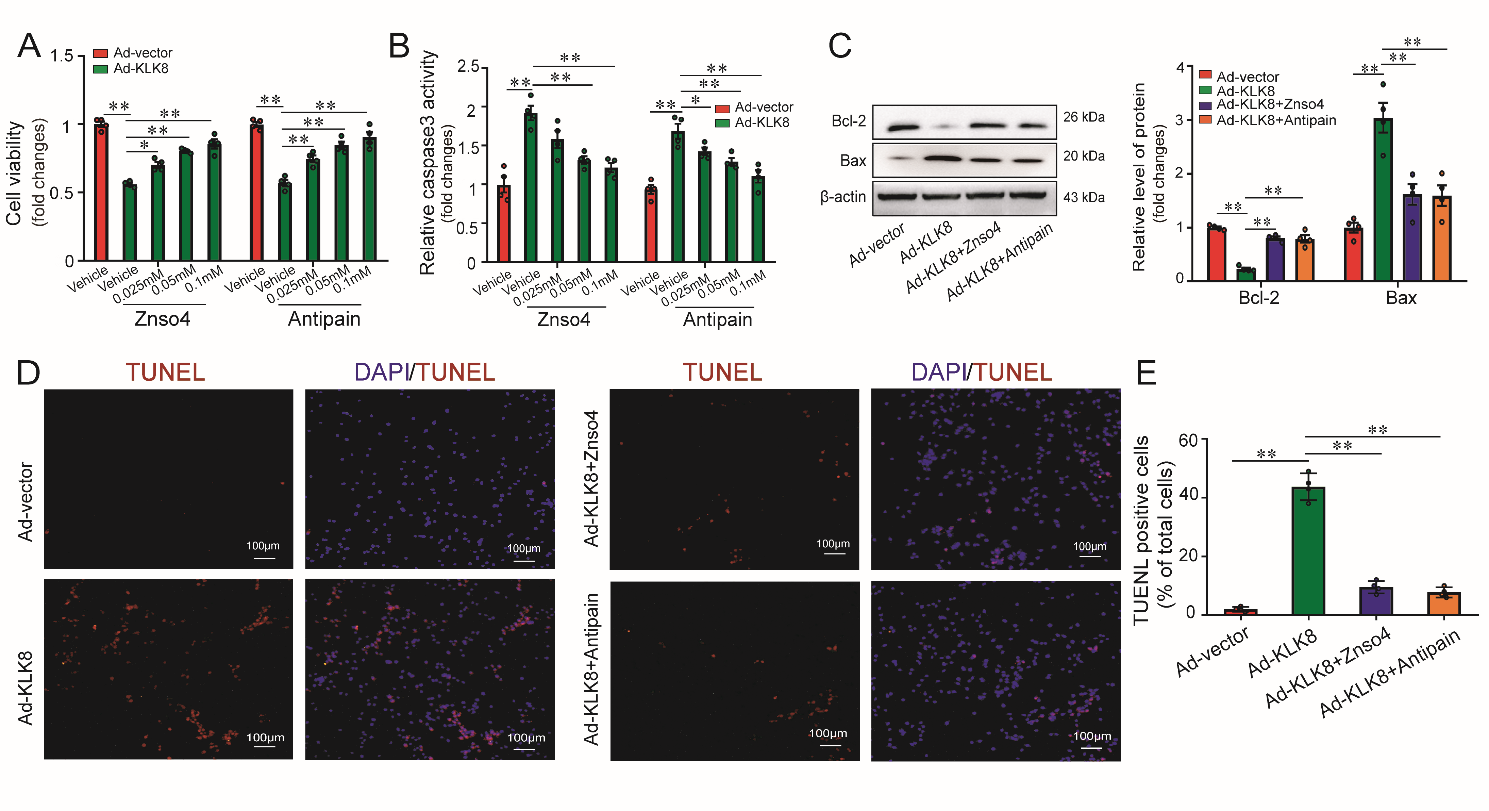


**Supplemental Fig. S4 Serine protease inhibitor rescues KLK8-overexpressed neuron** **cells from apoptosis.** HT22 murine hippocampal neuronal cells were infected with KLK8 adenovirus (Ad-KLK8) at a multiplicity of infection (MOI) of 3 for 24 h with or without two serine protease inhibitors, ZnSO4 and antipain. A and B, CCK8 assay and measurement of caspase-3 activity showed that both ZnSO4 and antipain blocked Ad-KLK8-induced cell injury and caspase-3 activation in a dose-dependent manner in HT22 cells, respectively. C, protein levels of Bcl-2 and Bax were determined by western blot analysis. The representative protein bands (left) and the corresponding histograms (right) showed that both ZnSO4 (0.05 mM) and antipain (0.05 mM) reversed Ad-KLK8-induced changes in protein levels of Bcl-2 and Bax. D, showed representative TUNEL-stained (red) cells. Nuclei were counterstained with DAPI (blue). Scale bar = 100 μm. E, the TUNEL-positive cell number (%) was shown as a ratio of the number of TUNEL-positive cells to the total cell number. D and E showed that both ZnSO4 (0.05 mM) and antipain (0.05 mM) reversed Ad-KLK8-induced HT22 cell apoptosis. Data were presented as means ± SD (n = 4, one-way ANOVA, Bonferroni’s post hoc test). * p < 0.05, ** p < 0.01.


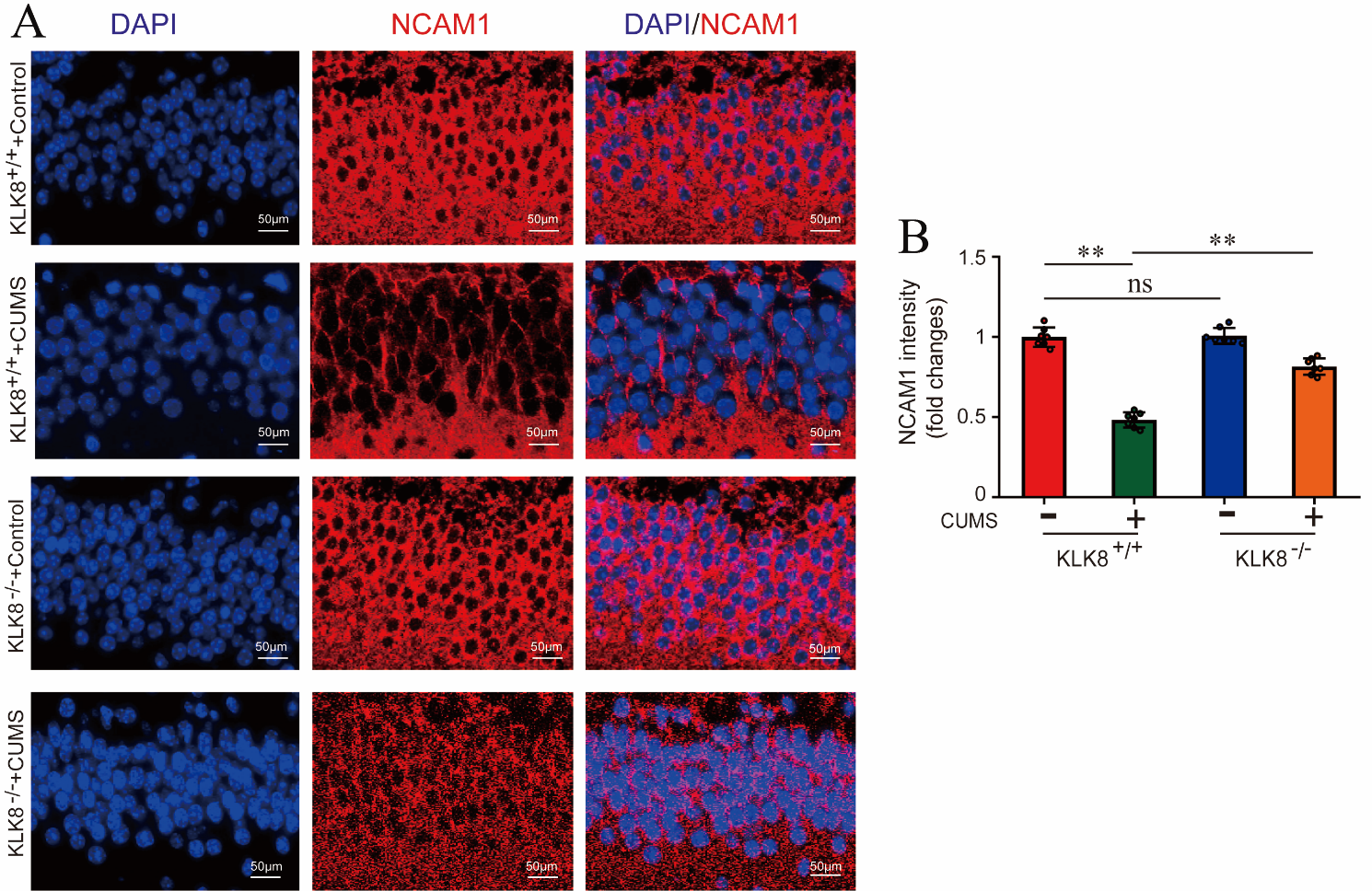


**Supplemental Fig. S5 The CUMS-induced loss of NCAM1 was largely prevented in KLK8-deficient mice.** A, immunofluorescent staining showed NCAM1 (red) levels in the cryostat-cut hippocampal sections of KLK8-/- mice. Nuclei were counterstained with DAPI (blue). Scale bar = 50 μm. B, showed quantification of the fluorescence intensity of the NCAM1 (n = 7, two-way ANOVA, Bonferroni’s post hoc test). ** p < 0.01, ns, not significant.


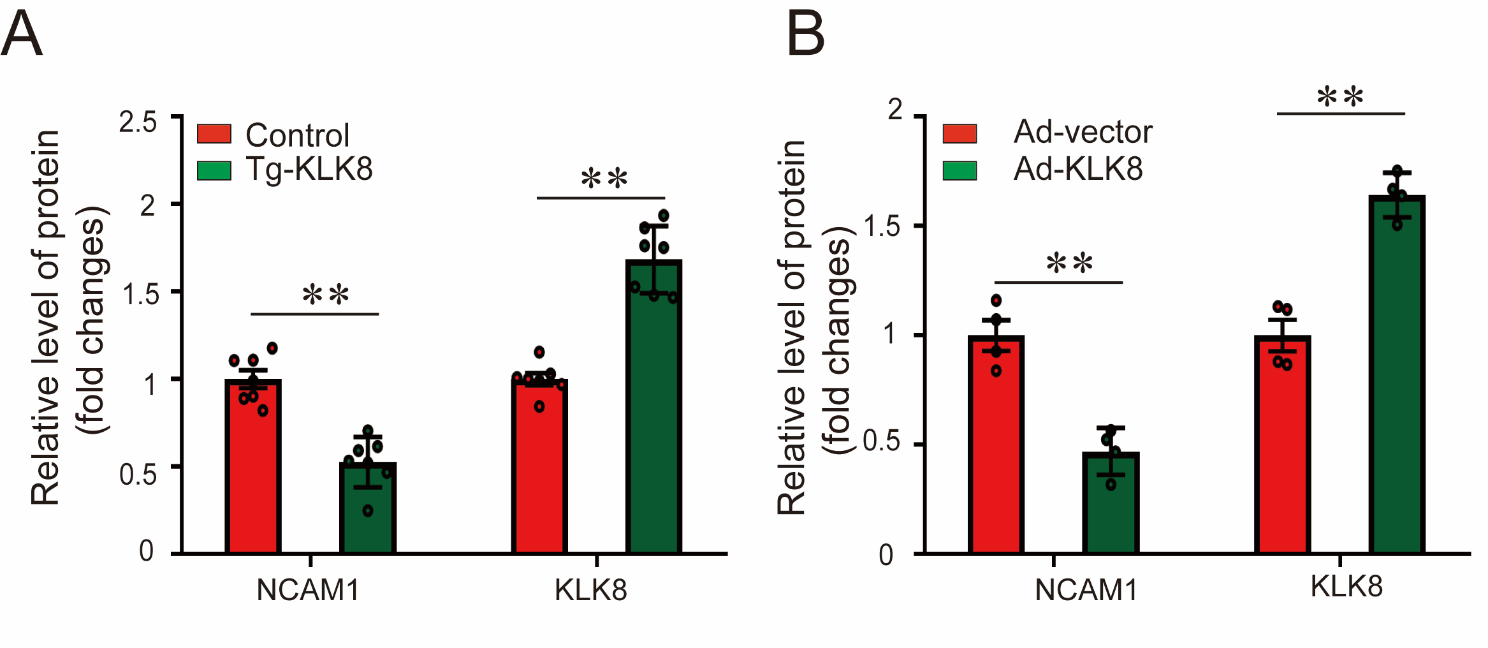


**Supplemental Fig. S6 KLK8 overexpression led to downregulation of NCAM1 in hippocampus and HT22 cells (related to Fig. 6).** A, showed relative densitometry of the NCAM1 and KLK8 protein levels in the hippocampus of KLK8 transgenic (Tg-KLK8) rats (n=7, unpaired t-test). B, showed relative densitometry of the NCAM1 and KLK8 protein levels in KLK8 adenovirus (Ad-KLK8)-treated HT22 cells (n=4, unpaired t-test). Data were presented as means ± SD. ** p < 0.01.


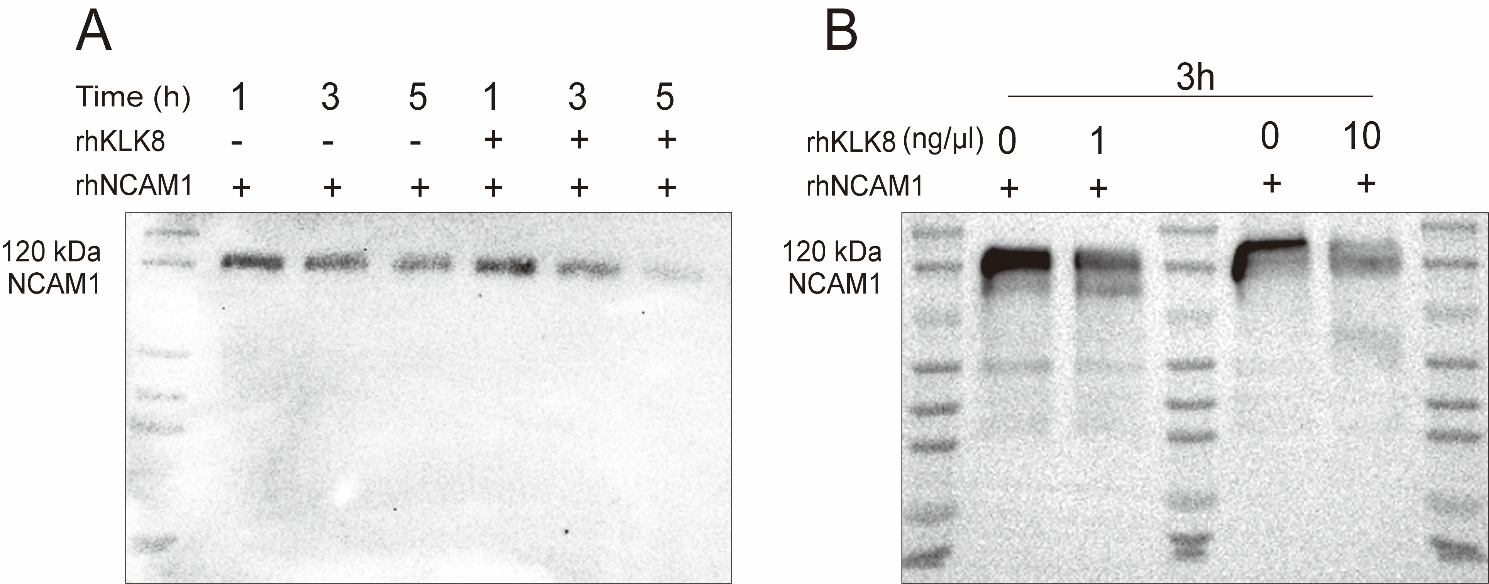


**Supplemental Fig. S7 KLK8 directly cleaves NCAM1.** A, purified recombinant NCAM1 (1 ng/μl) was incubated with or without activated recombinant KLK8 (1 ng/μl) for the indicated time periods, and analyzed by western blot using NCAM1 antibody. B, purified recombinant NCAM1 (1 ng/μl) was incubated with or without activated recombinant KLK8 at the indicated doses, and analyzed by western blot using NCAM1 antibody.


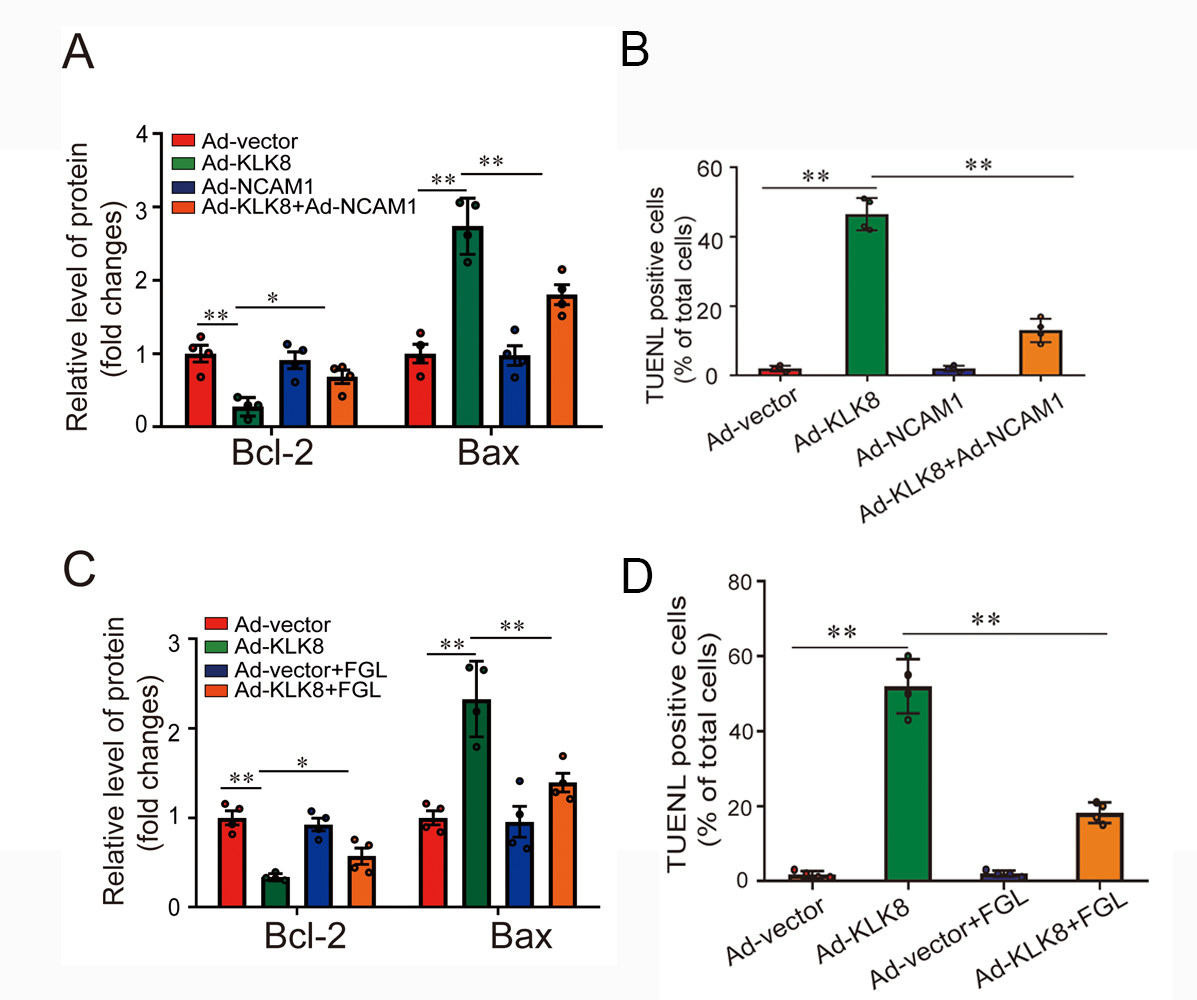


**Supplemental Fig. S8 Both NCAM1 overexpression and NCAM1 mimetic peptide rescue KLK8-overexpressed HT22 cells from apoptosis (related to Fig. 7).** HT22 murine hippocampal neuronal cells were infected with KLK8 adenovirus (Ad-KLK8) at a multiplicity of infection (MOI) of 3 for 24 h with or without NCAM1 adenovirus (Ad-NCAM1) (A-B) or NCAM1 mimetic peptide FGL (C-D). A and C, showed relative densitometry of the Bcl-2 and Bax protein levels. B and D, the TUNEL-positive cell number (%) was shown as a ratio of the number of TUNEL-positive cells to the total cell number. Data are expressed as means ± SD (n = 4, one-way ANOVA, Bonferroni’s post hoc test). * p < 0.05, ** p < 0.01.
