# Supplementary material for: Upregulation of KLK8 contributes to CUMS-induced hippocampal neuronal apoptosis by cleaving NCAM1
Source: Cell Death Dis. 2023 Apr 19;14(4):278. doi: 10.1038/s41419-023-05800-5 (PMC10115824; doi:10.1038/s41419-023-05800-5)

Figure.1E

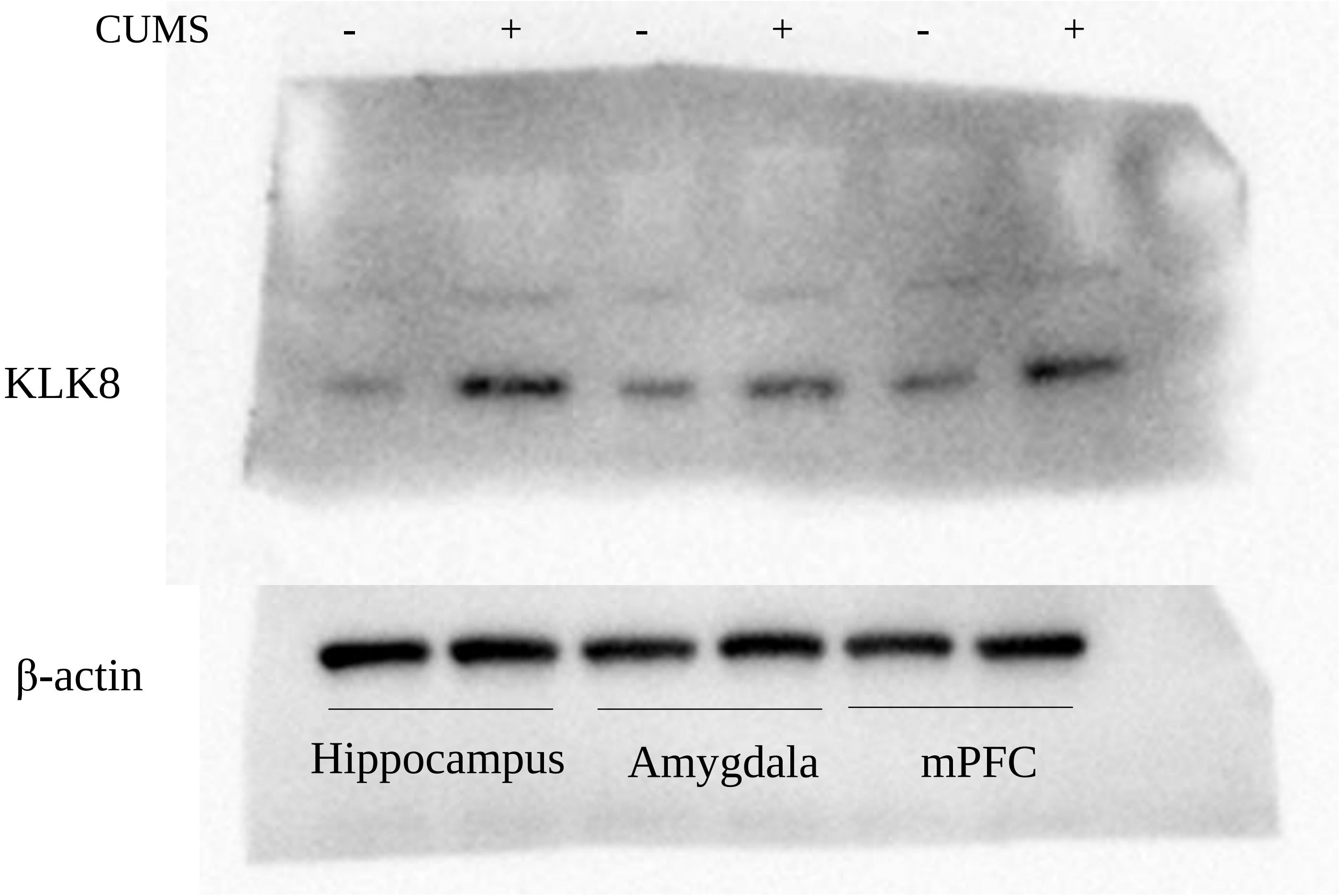

Figure.2A

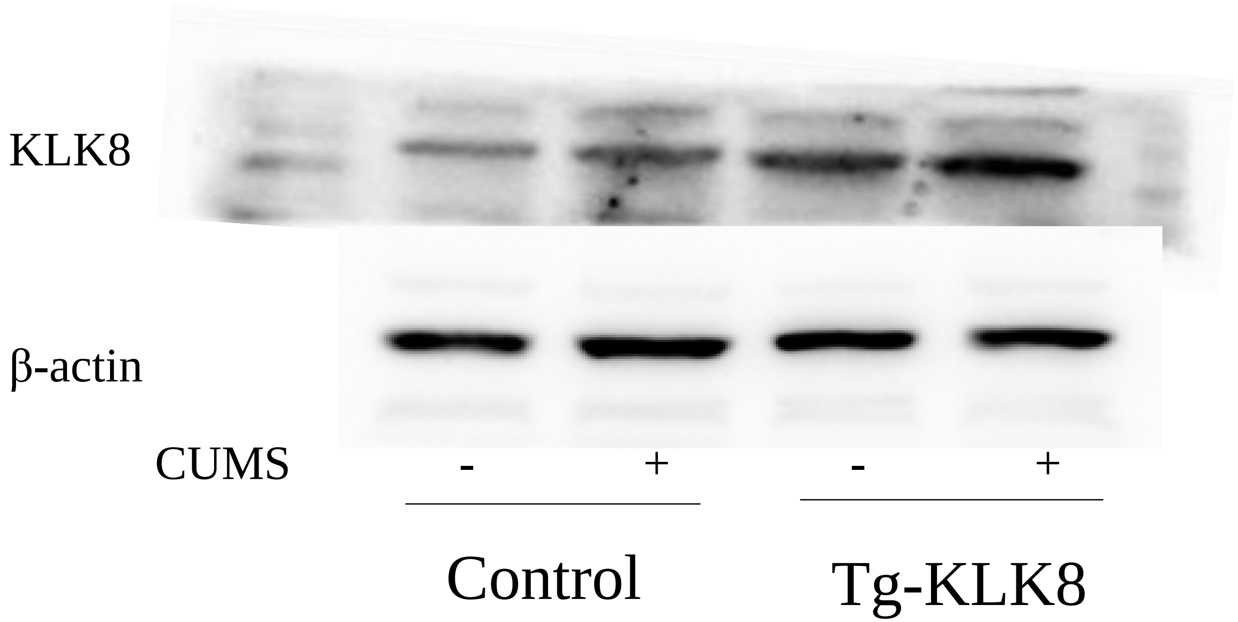

Figure.2E

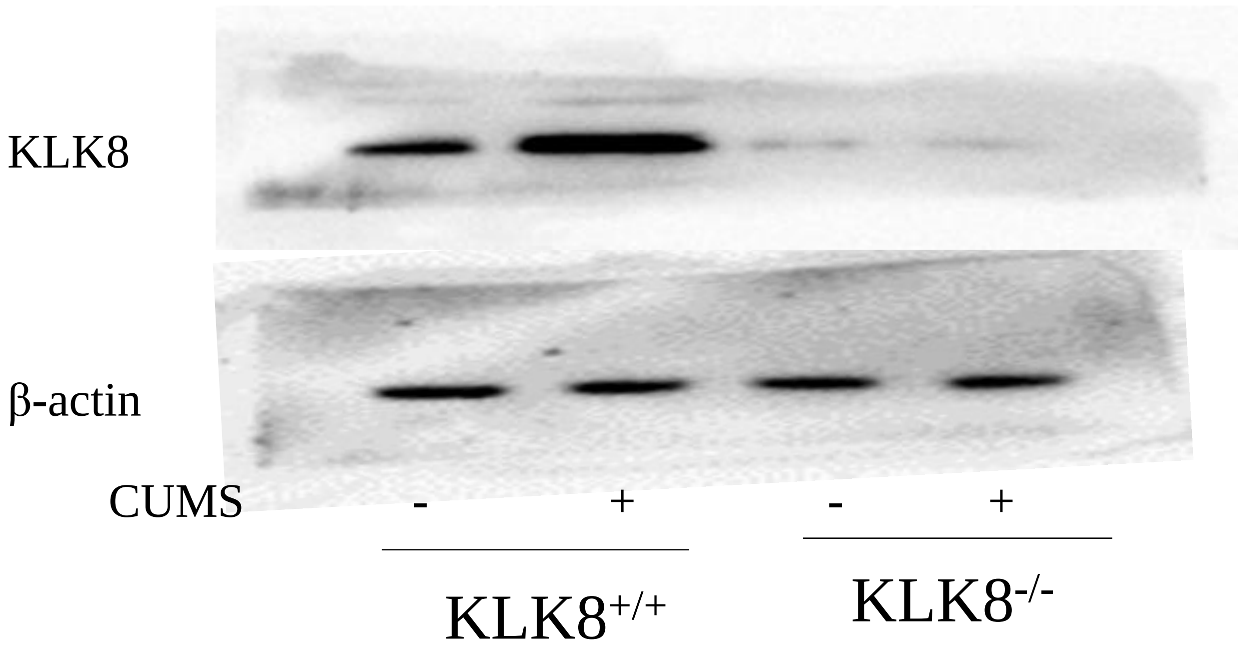

Figure.3A

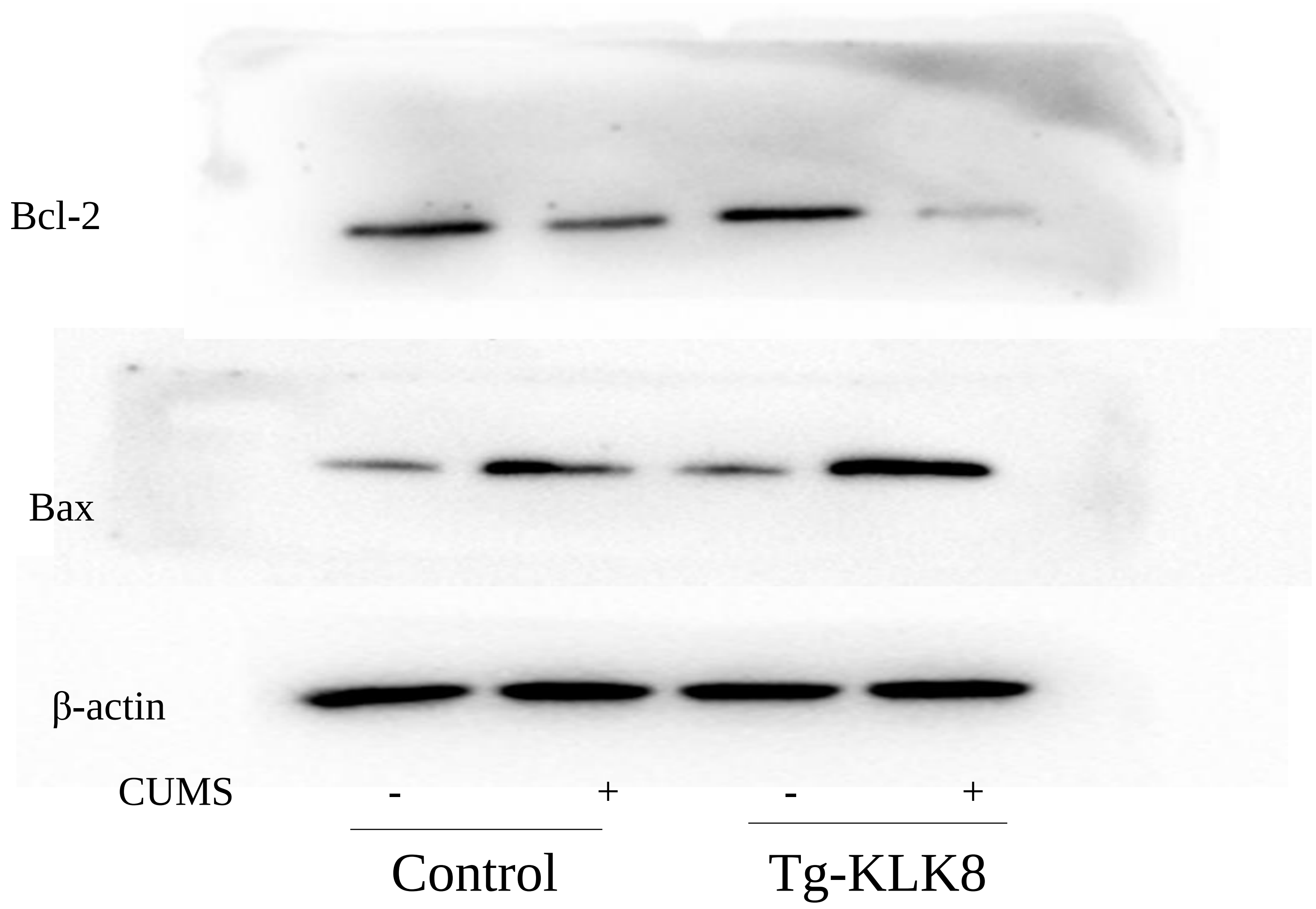

Figure.3D

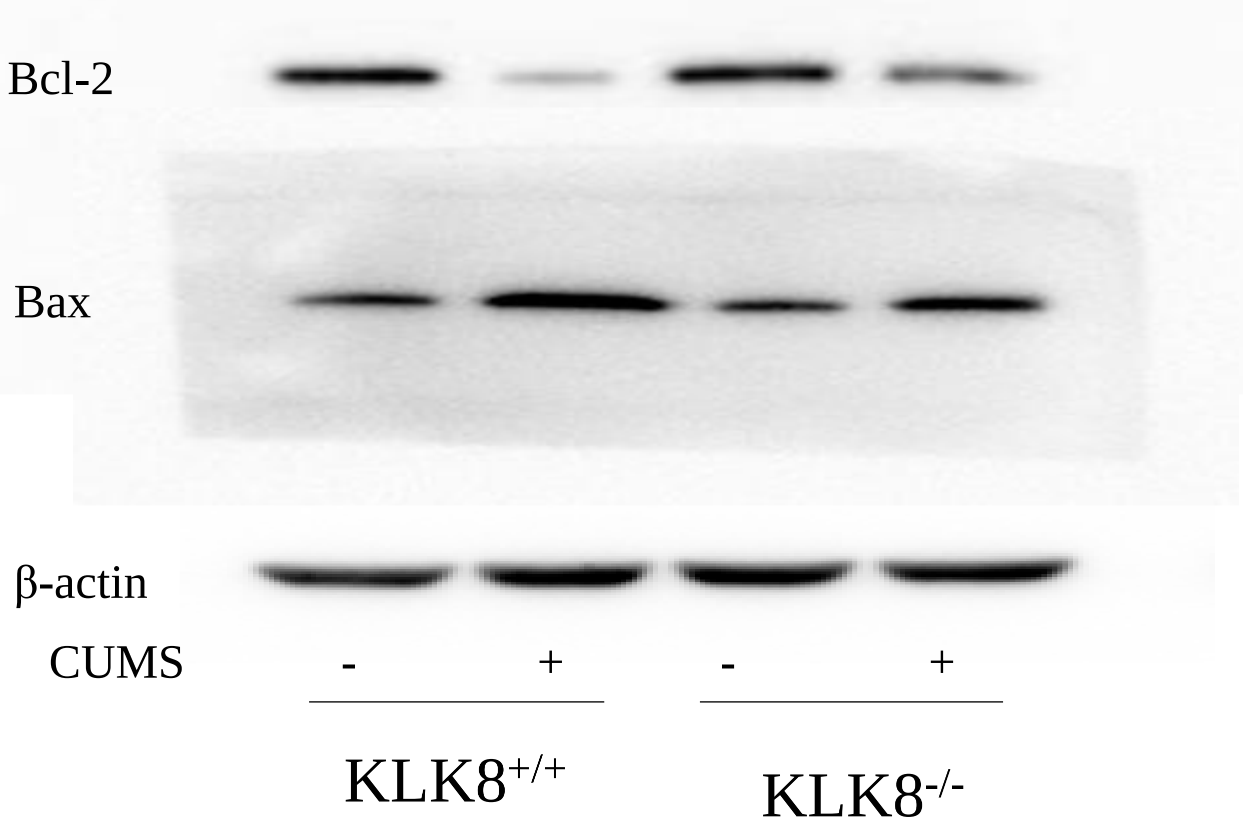

Figure.4A

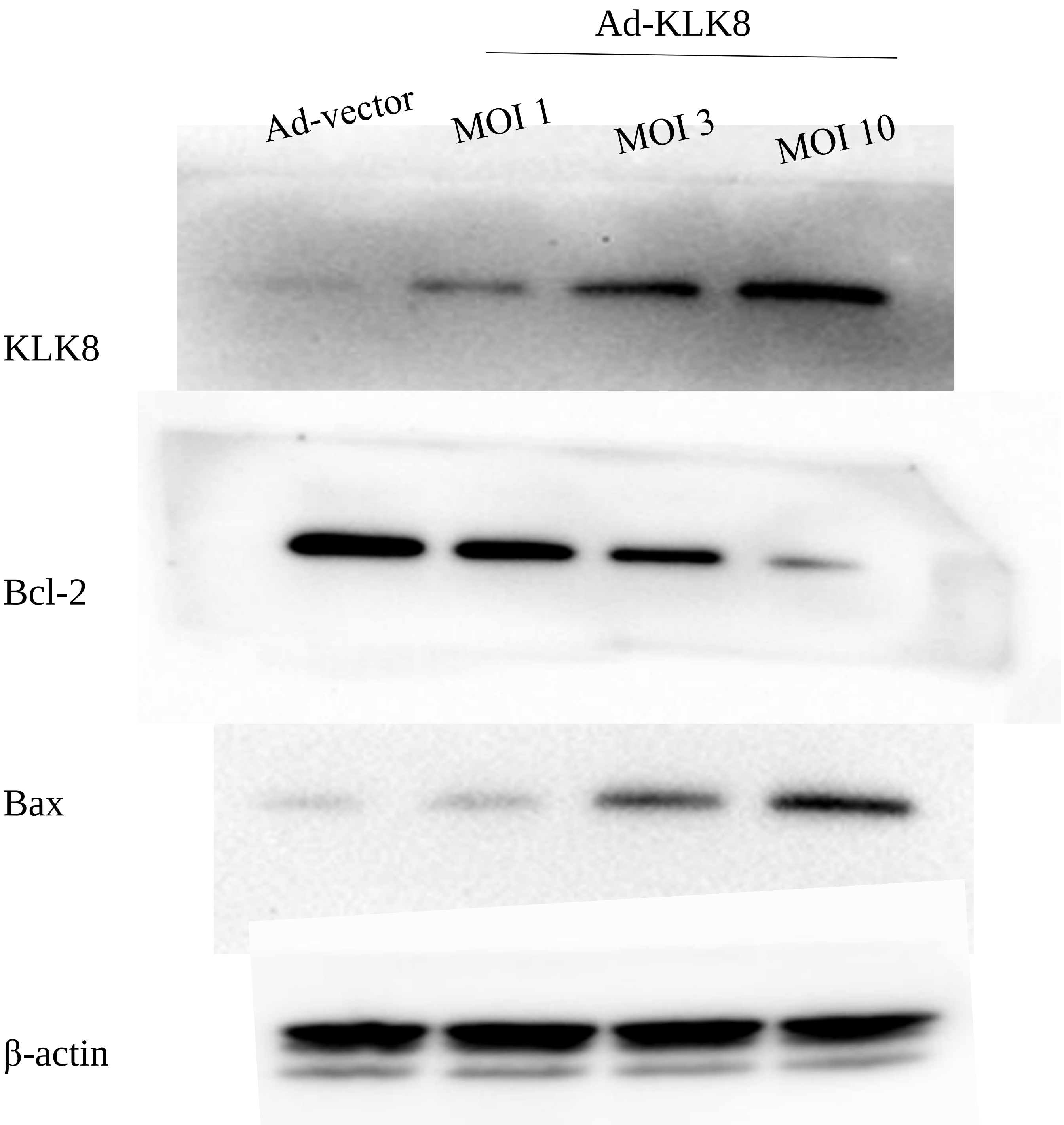

Figure.4E

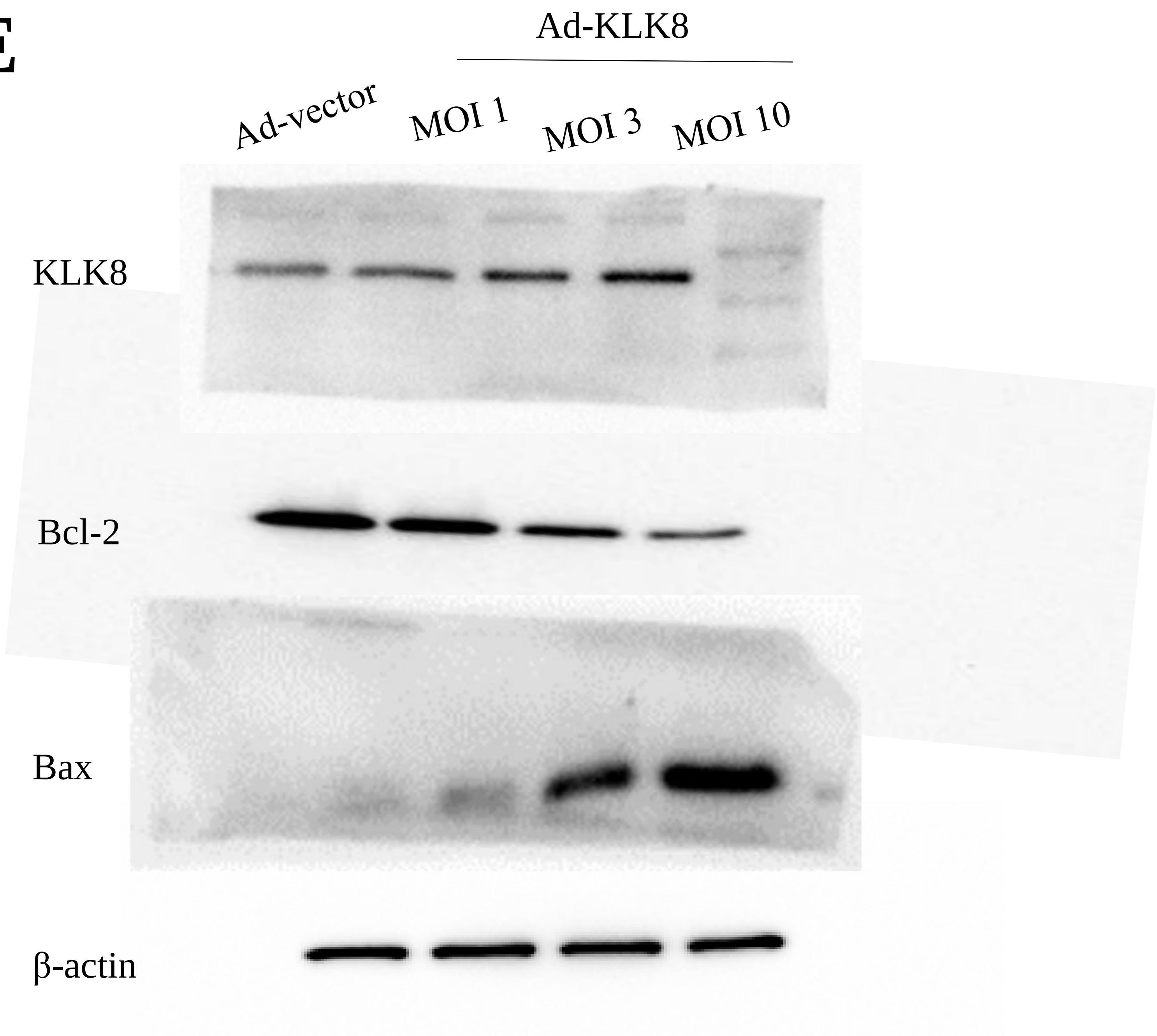

Figure.5C

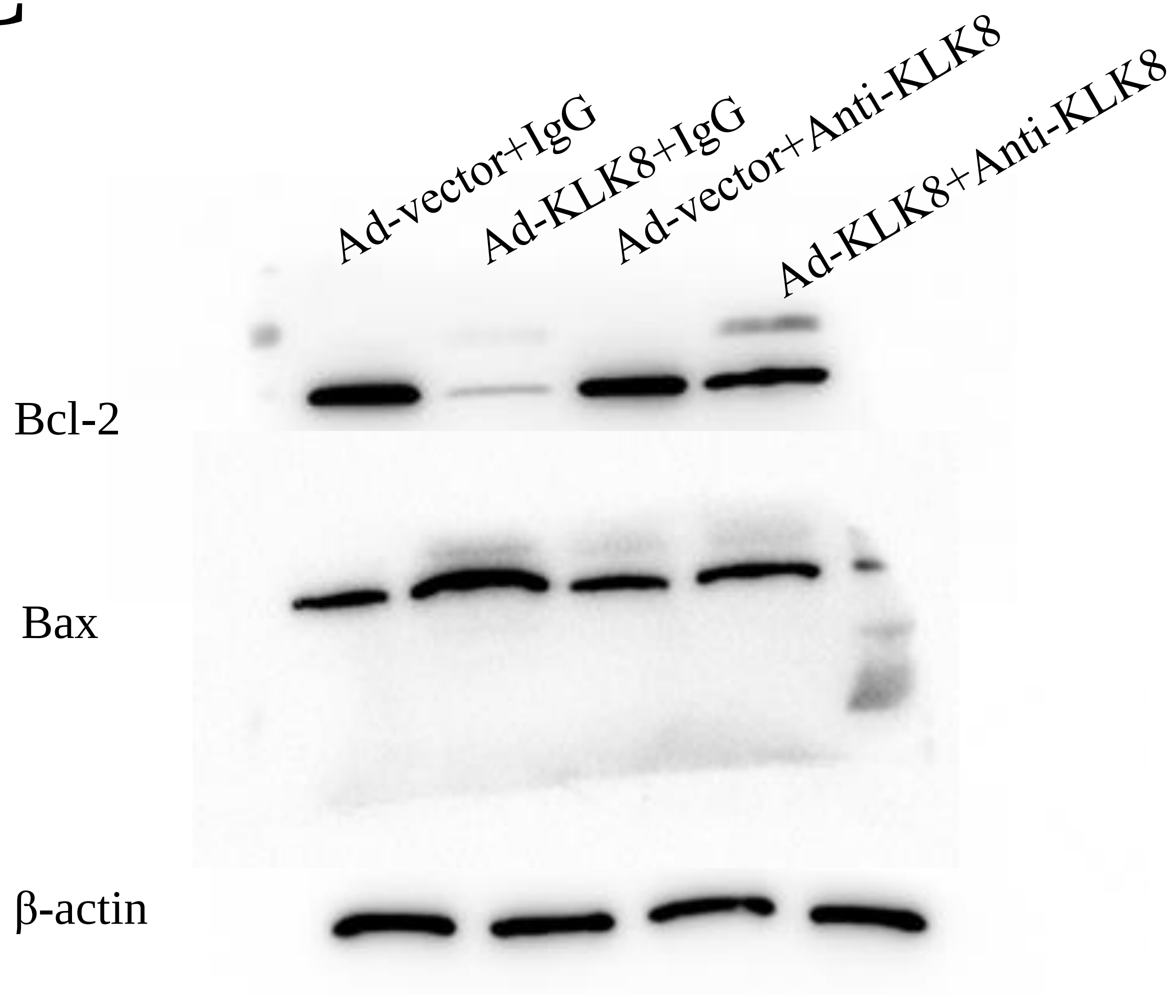
Ad-vector+IgG
Ad-KLK8+IgG
Ad-vector+Anti-KLK8
Ad-KLK8+Anti-KLK8
Bcl-2
Bax
β-actin

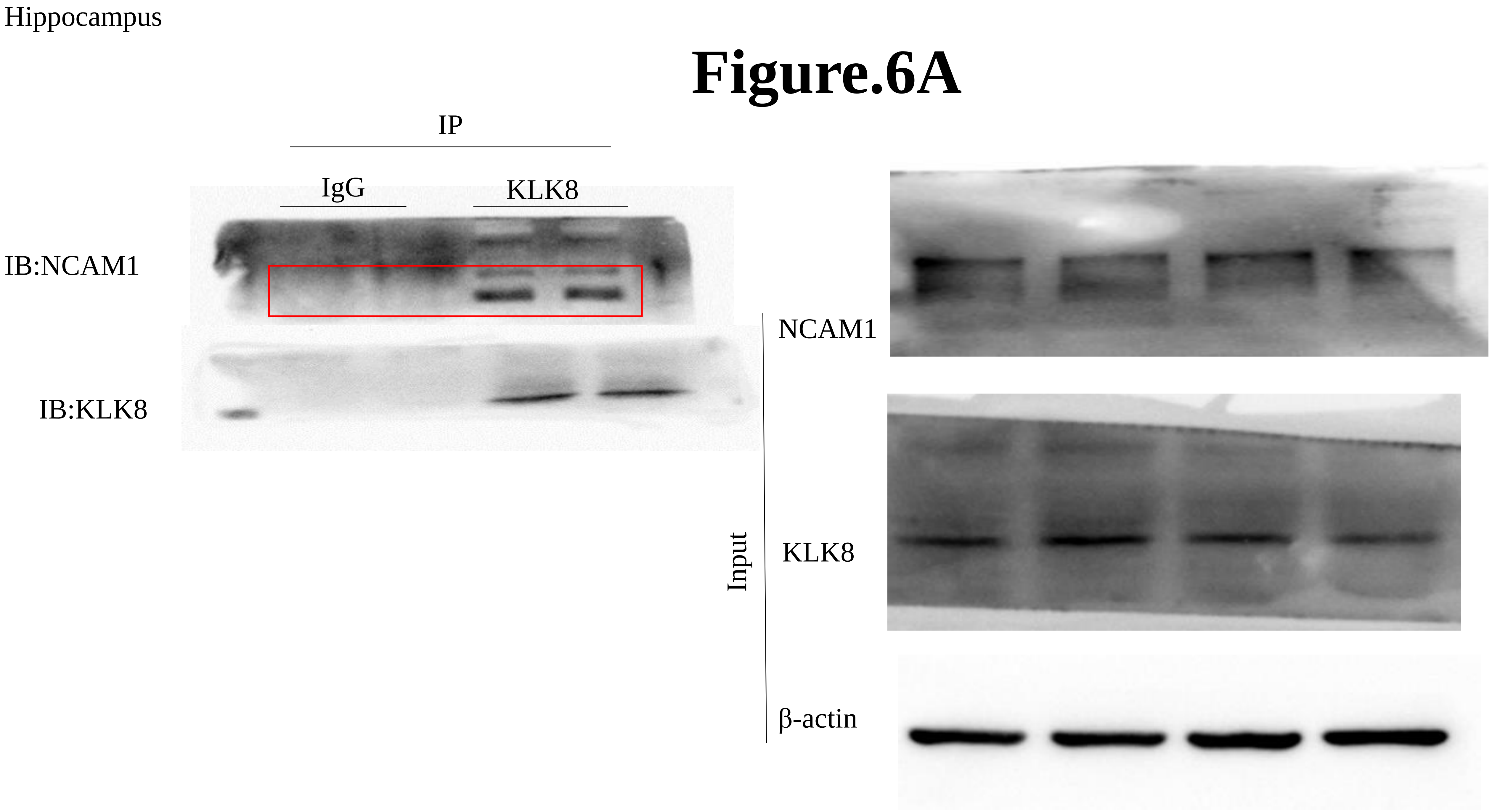

Hippocampus
Figure.6A
IP
IgG
KLK8
IB:NCAM1
IB:KLK8
Input
NCAM1
KLK8
β-actin

HT22

Figure.6A

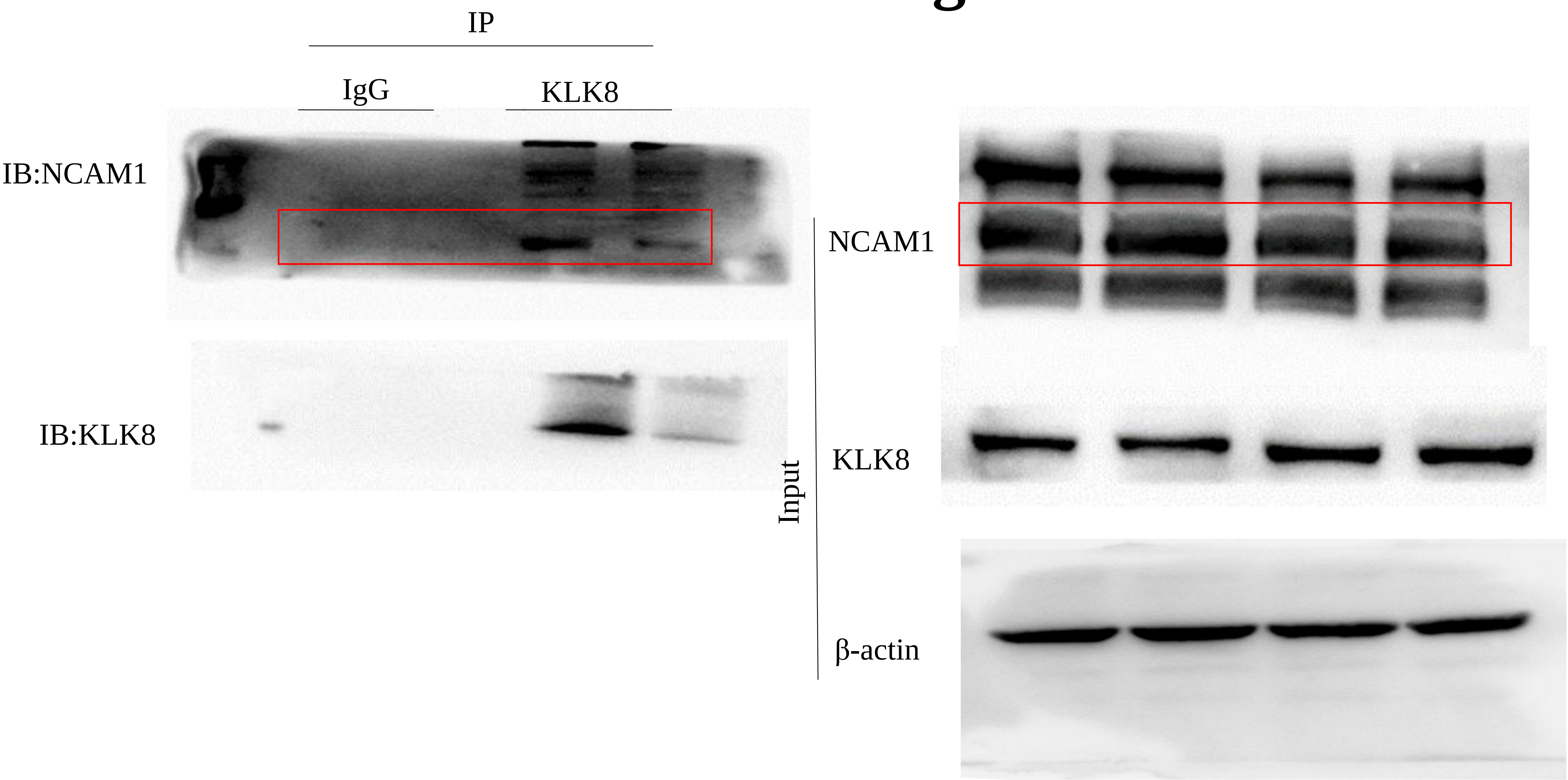

Hippocampus

Figure.6A

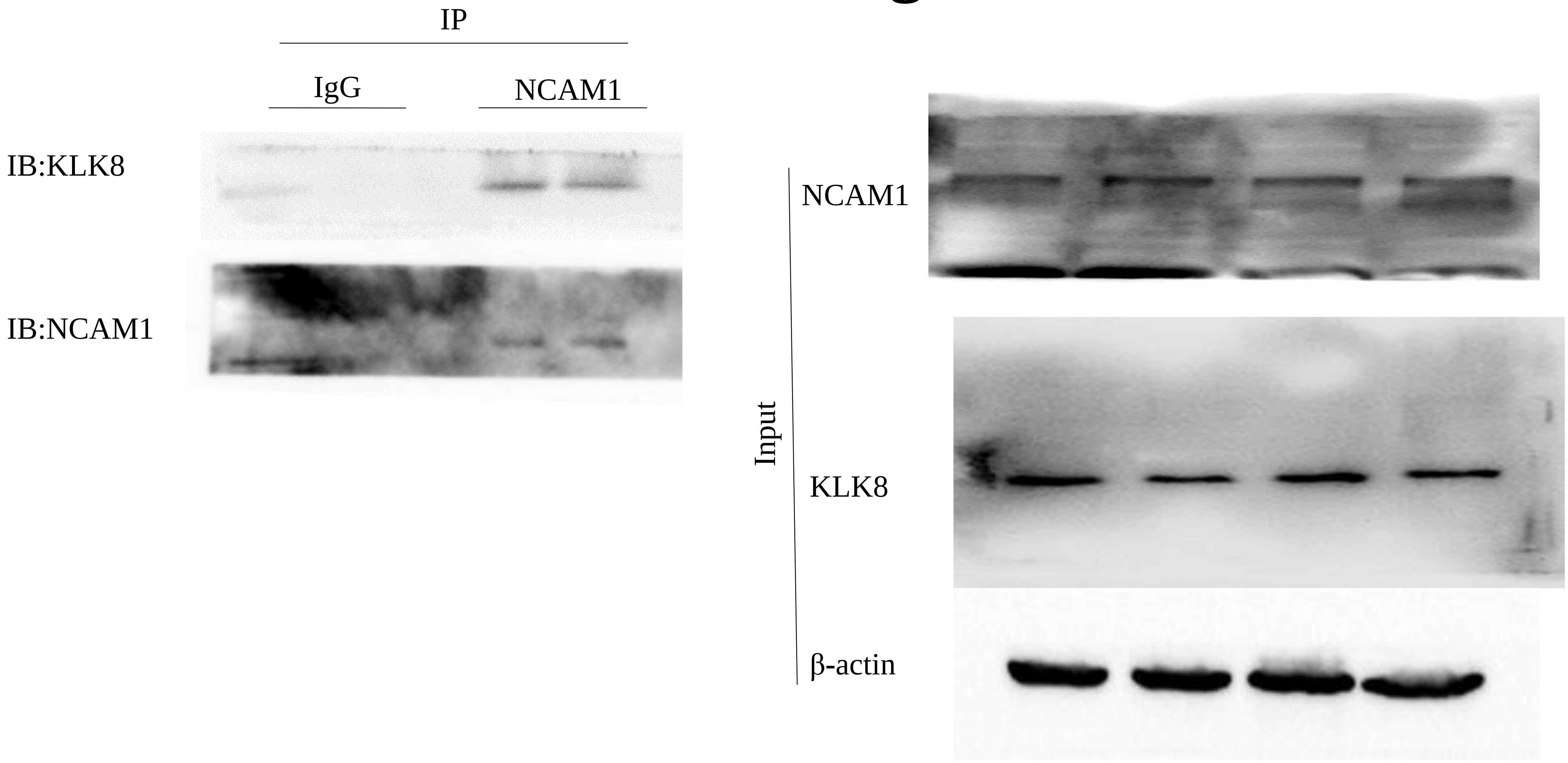

Figure.6A

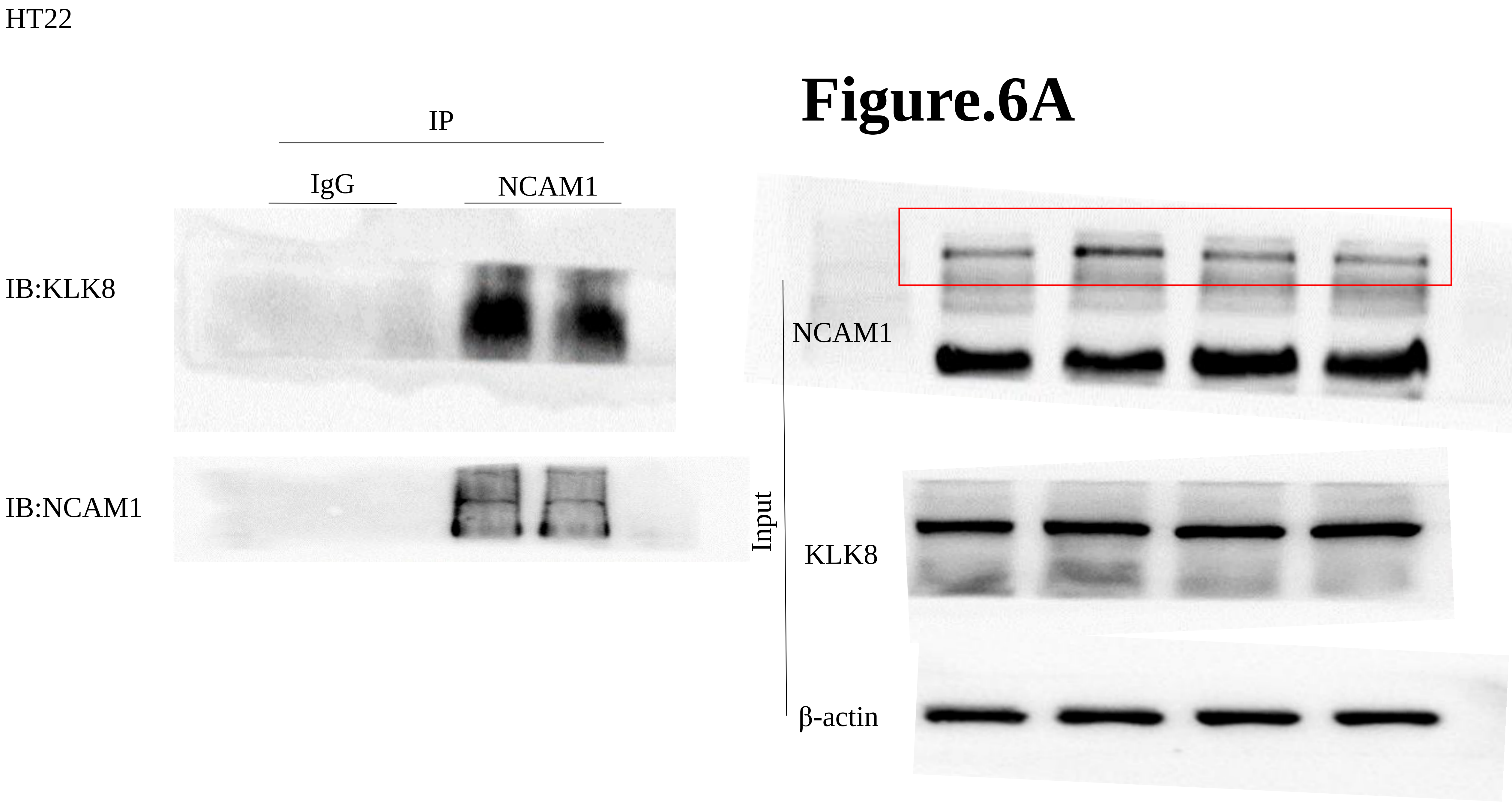

Figure.6D

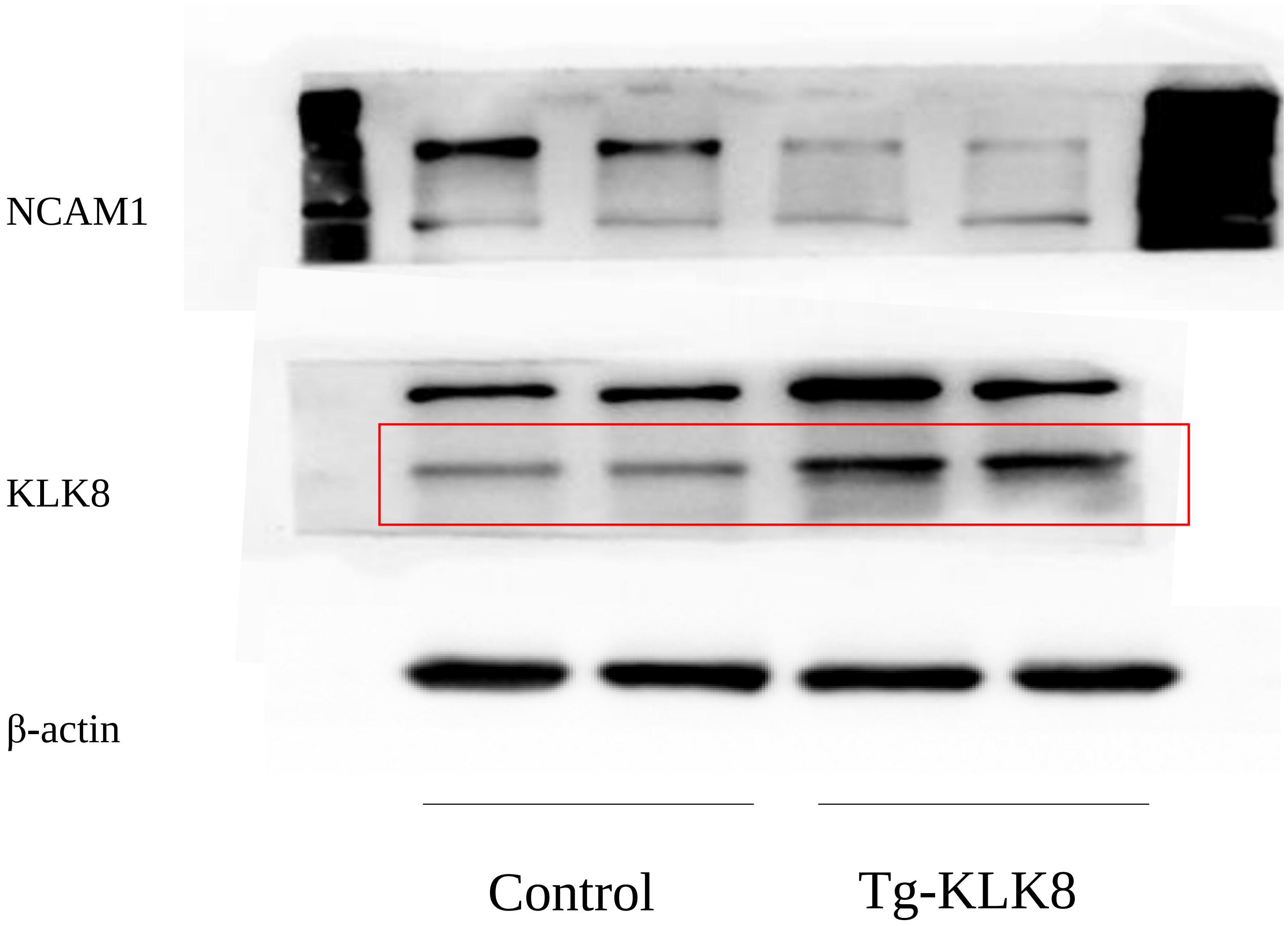

Figure.6E

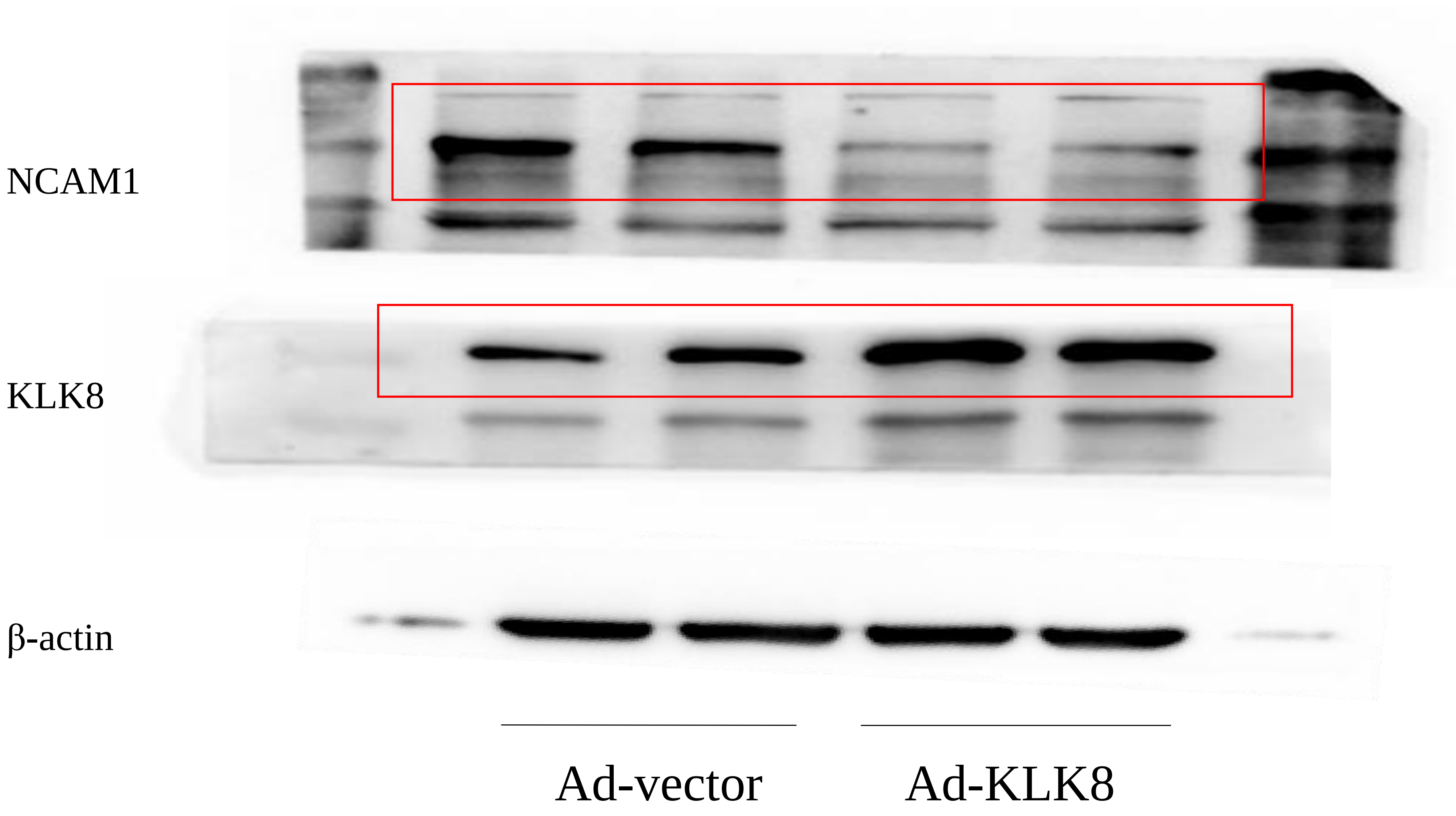

Medium

Figure.6F

Ad-vector Ad-KLK8

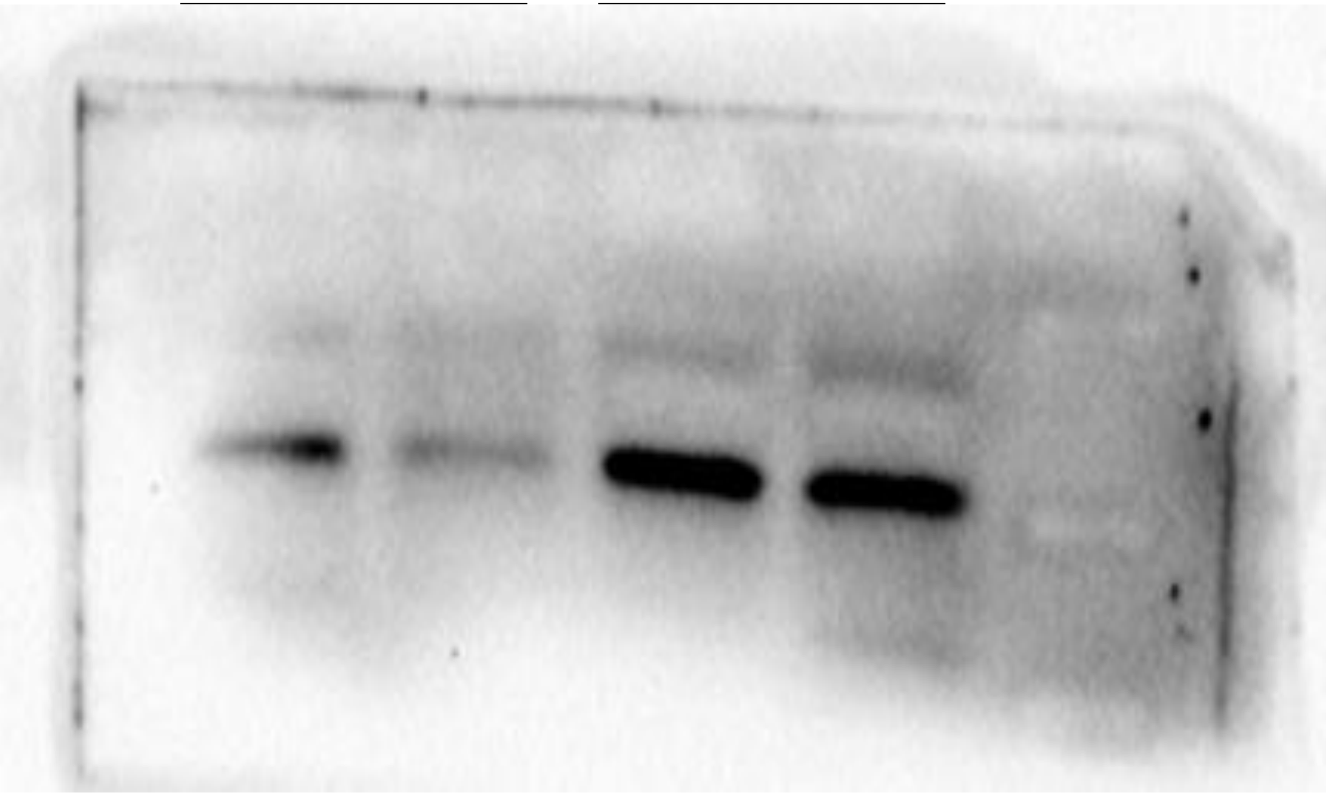

Ad-vector Ad-KLK8

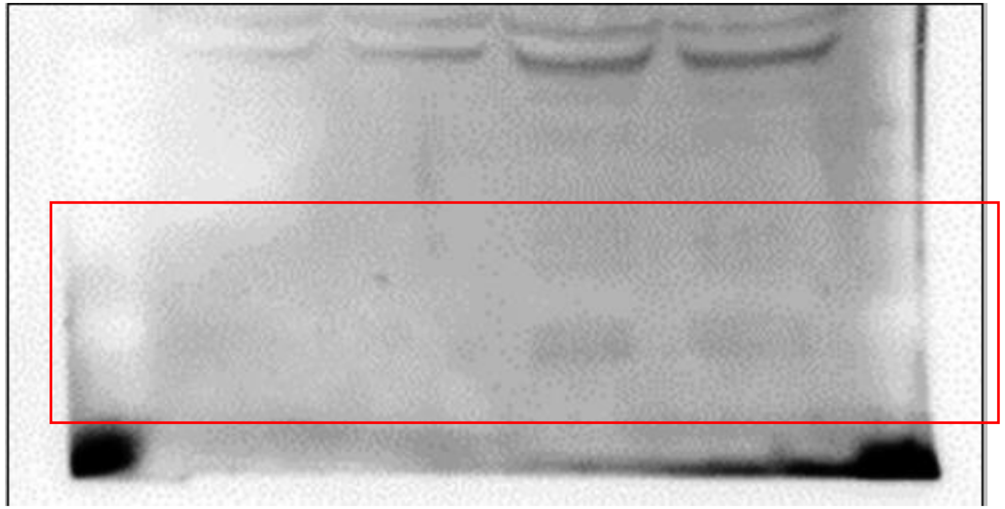

Figure.6F

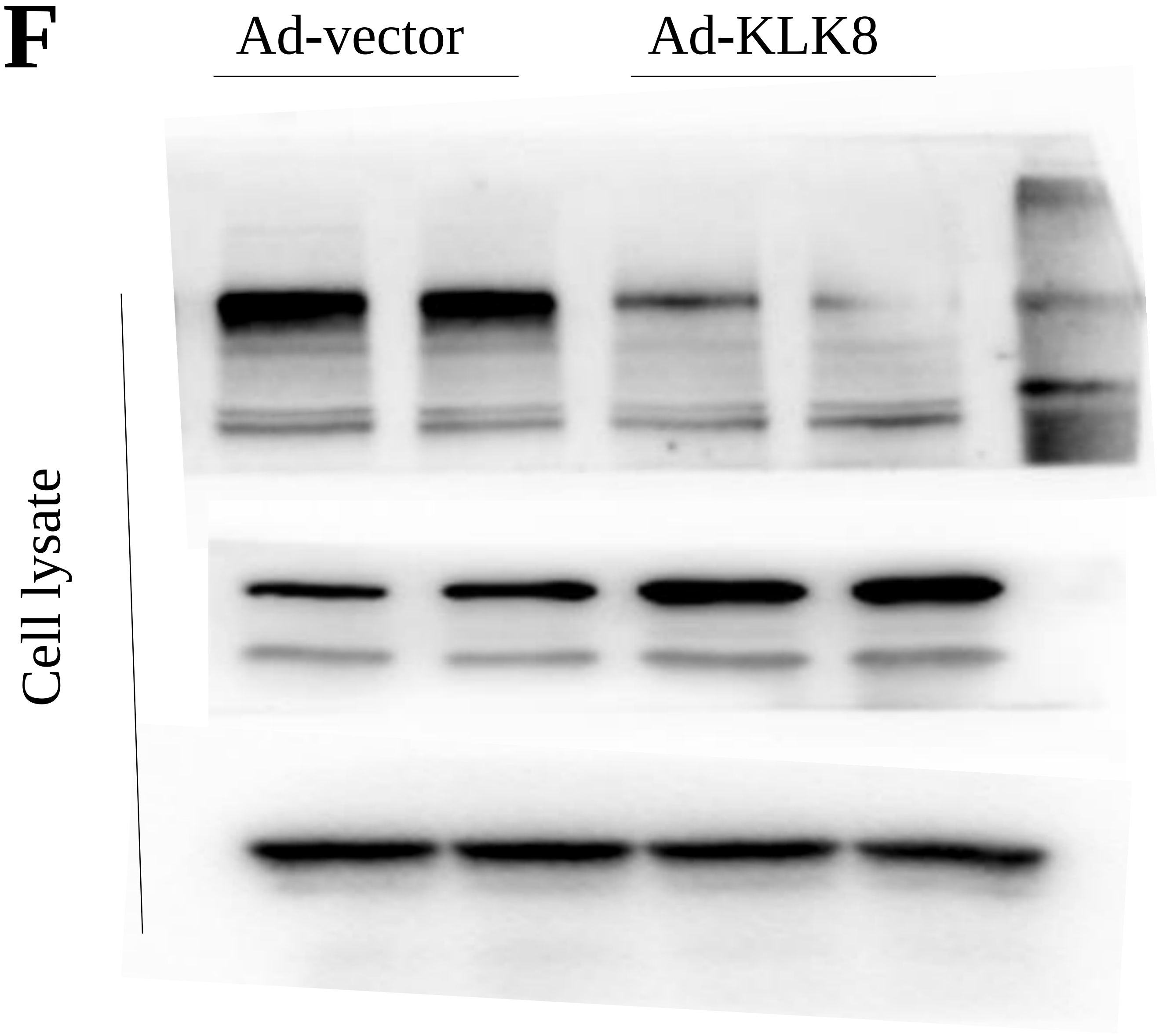

Figure.7C

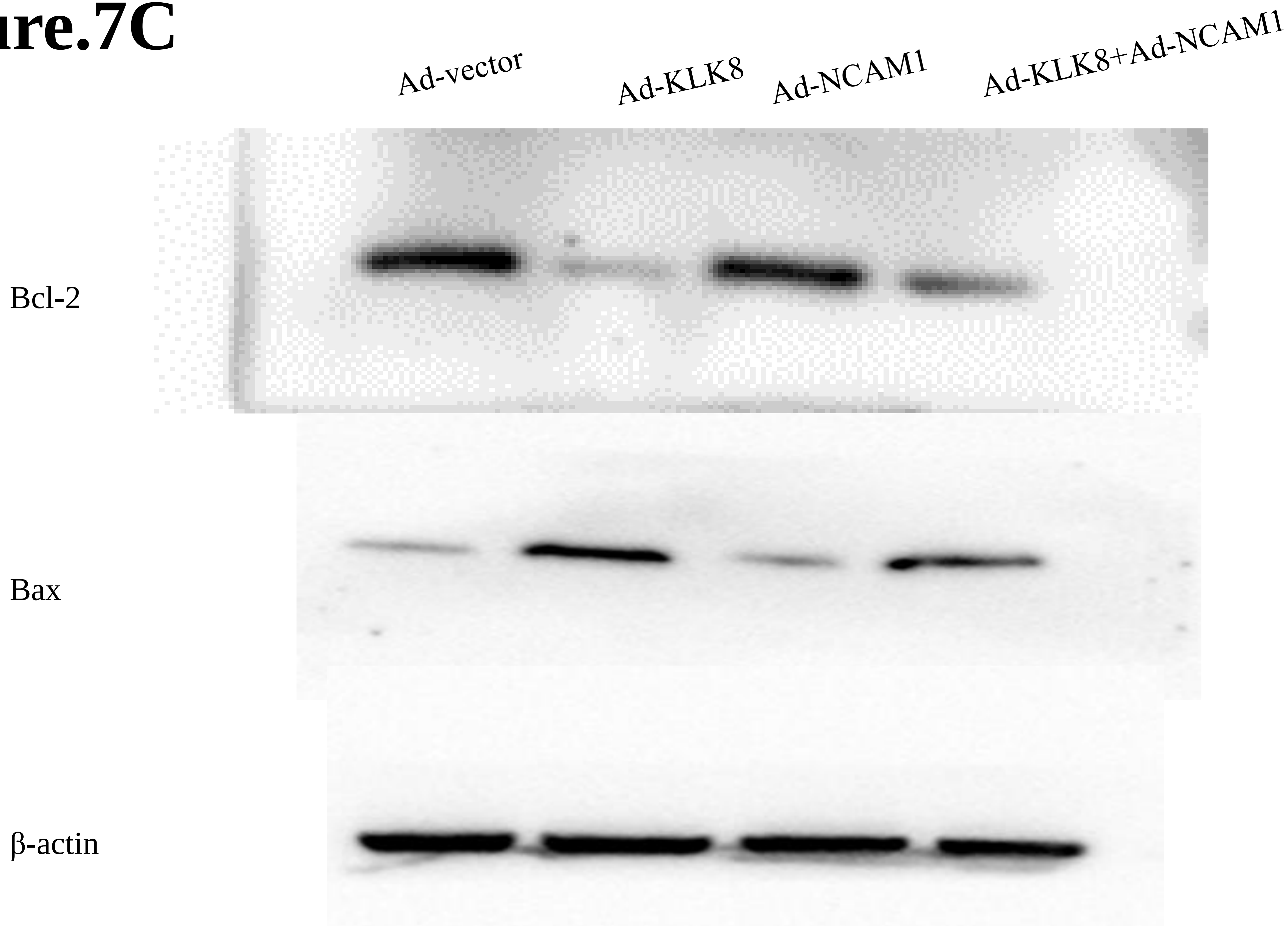

Figure.7G

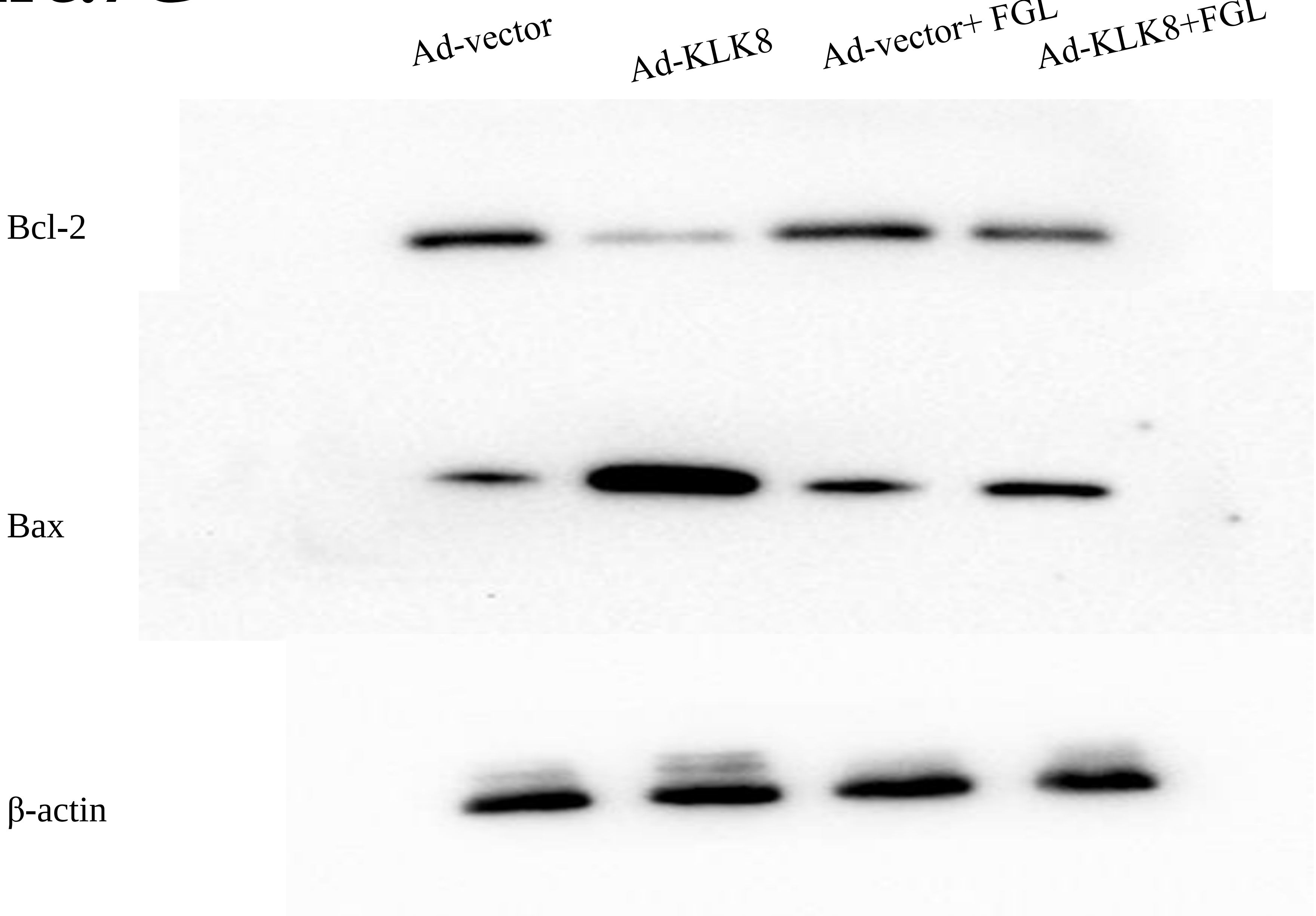

Figure.8C

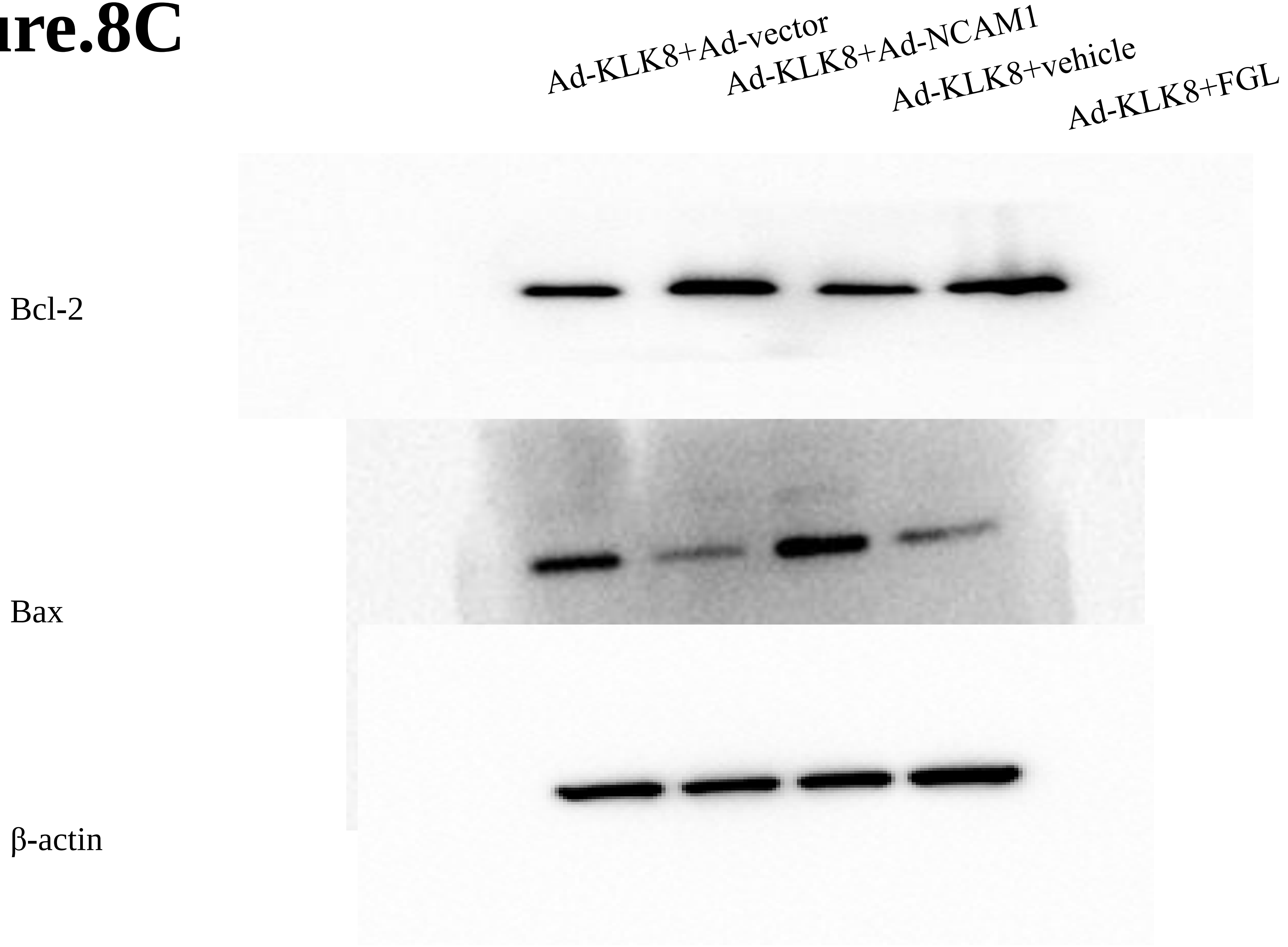
Ad-KLK8+Ad-vector
Ad-KLK8+Ad-NCAM1
Ad-KLK8+vehicle
Ad-KLK8+FGL
Bcl-2
Bax
β-actin

Supplemental Fig. S1A

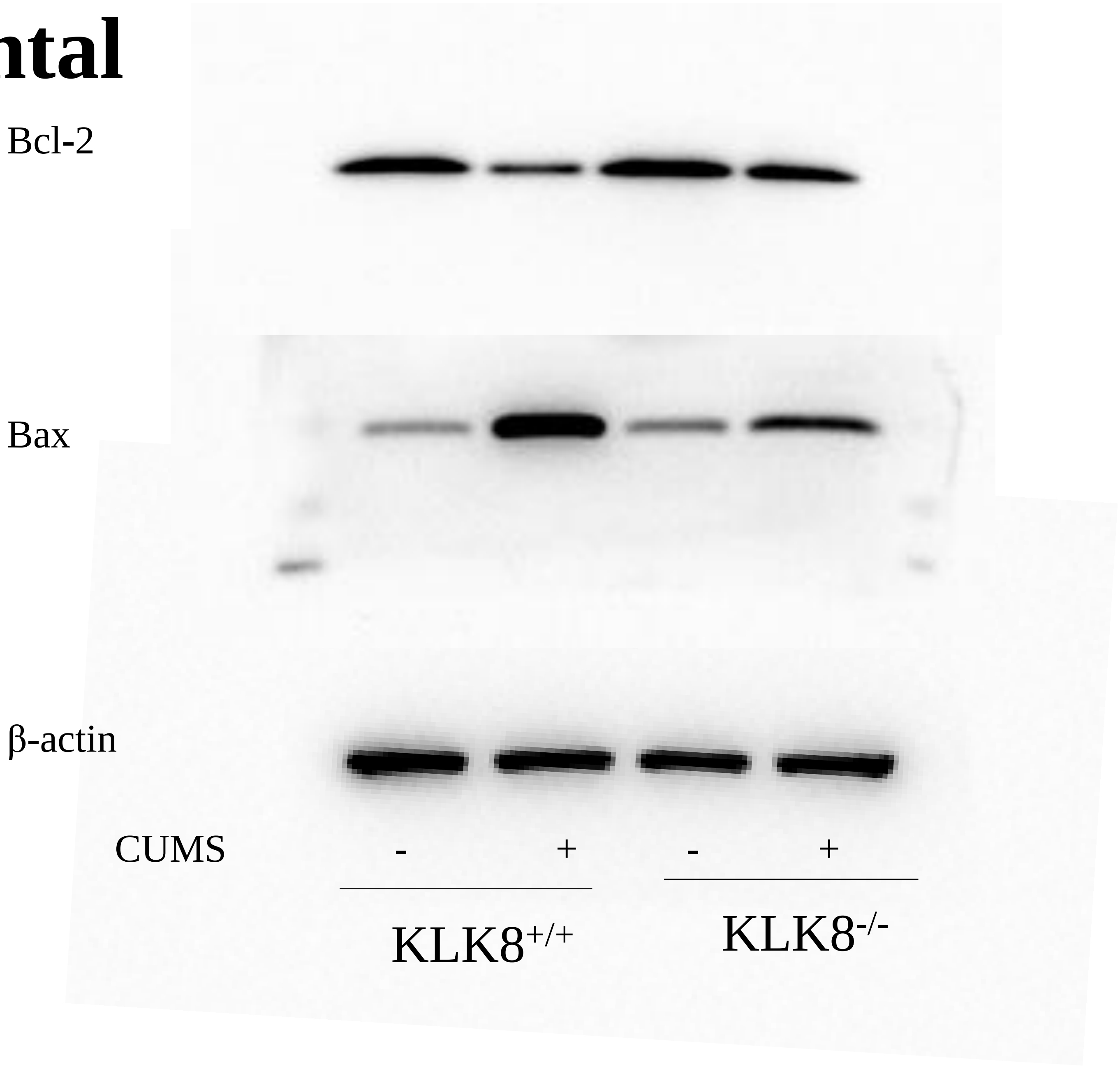

Supplemental Fig. S2A

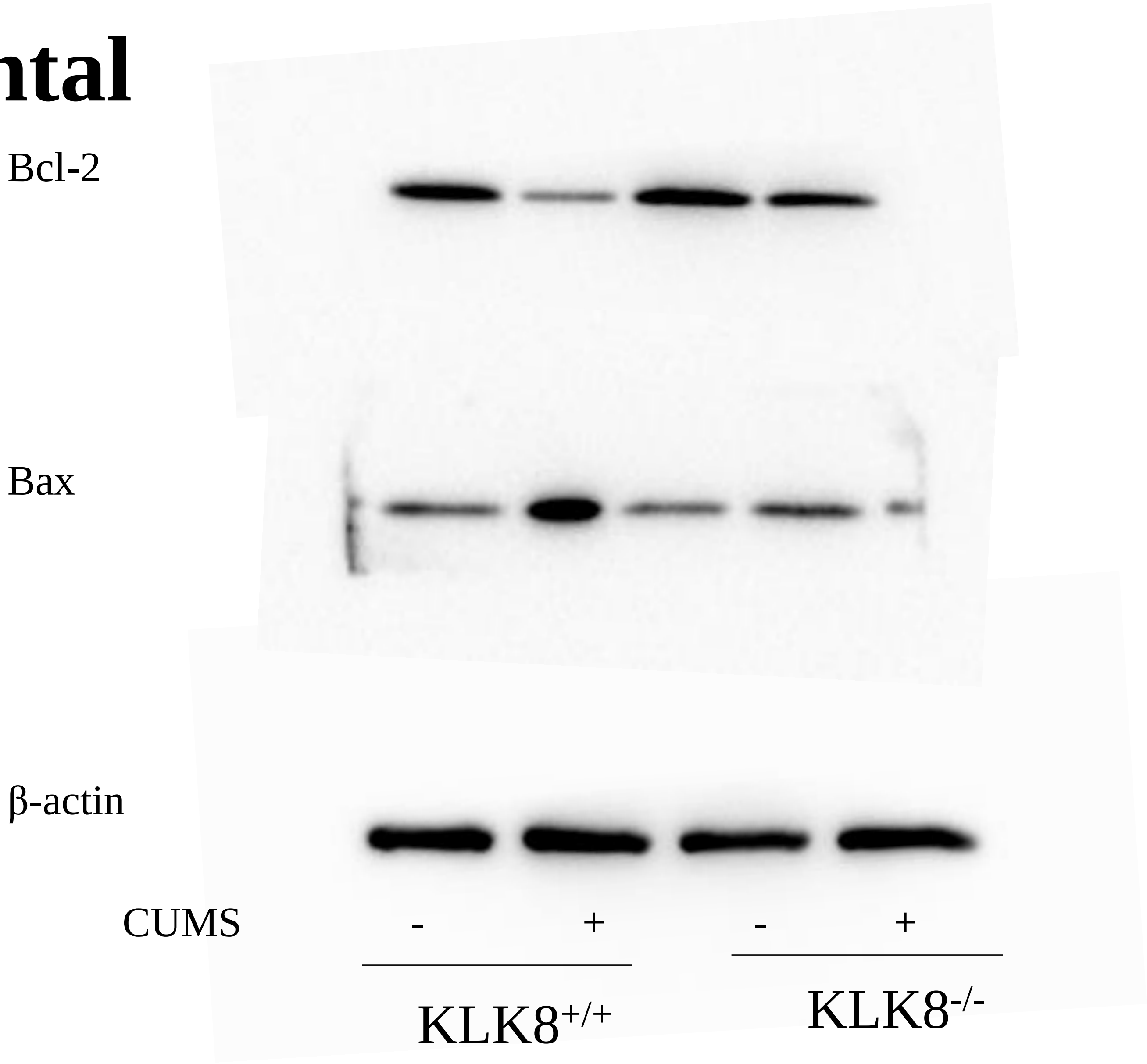

Supplemental Fig. S4C

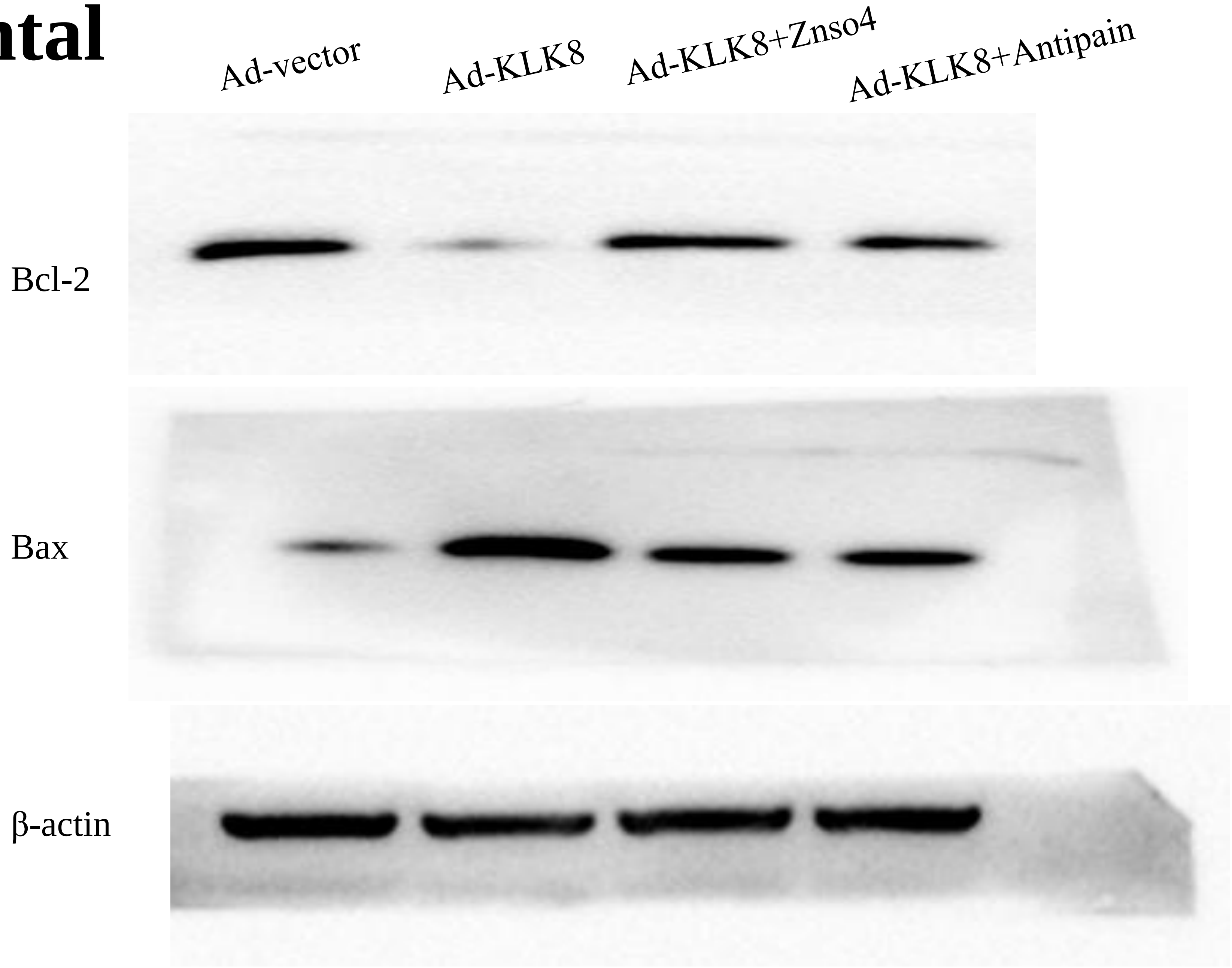

Supplemental Fig. S7A

Time (h)	1	3	5	1	3	5
rhKLK8	–	–	–	+	+	+
rhNCAM1	+	+	+	+	+	+

120 kDa NCAM1

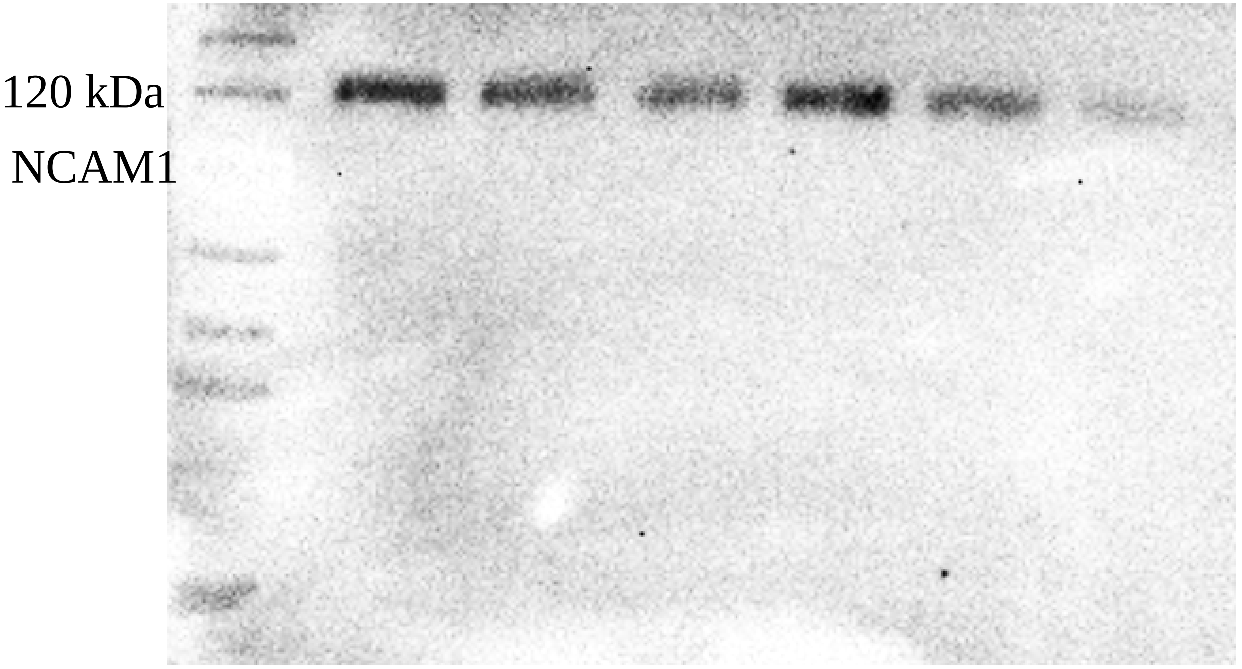

Supplemental Fig. S7B

3h

rhKLK8 (ng/ µl)	0	1	0	10
rhNCAM1	+	+	+	+

120 kDa NCAM1

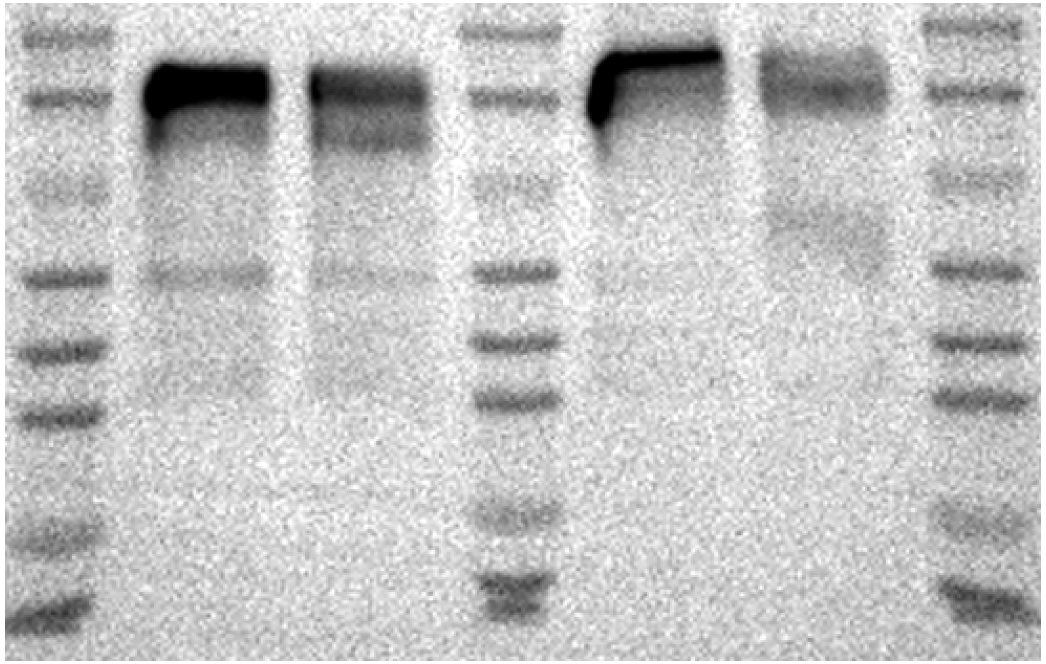

Supplement: Supplementary file 2 — suppleement material [file 41419_2023_5800_MOESM2_ESM.pdf]
